# Pharmacokinetic Adaptations in Pregnancy: Implications for Optimizing Antiretroviral Therapy in HIV-Positive Women

**DOI:** 10.3390/pharmaceutics17070913

**Published:** 2025-07-15

**Authors:** Natalia Briceño-Patiño, María Camila Prieto, Paula Manrique, Carlos-Alberto Calderon-Ospina, Leonardo Gómez

**Affiliations:** 1 School of Medicine and Health Sciences, Universidad del Rosario, Bogota 111221, Colombiacarlos.calderon@urosario.edu.co (C.-A.C.-O.); 2 Research Group in Applied Biomedical Sciences (UR Biomed), School of Medicine and Health Sciences, Universidad del Rosario, Bogota 111221, Colombia; 3 Center for Research in Genetics and Genomics (CIGGUR), Institute of Translational Medicine (IMT), School of Medicine and Health Sciences, Universidad Del Rosario, Bogota 111221, Colombia; 4 Departamento de Salud Sexual y Reproductiva, Hospital Universitario Mayor Mederi, Bogota 111411, Colombia

**Keywords:** pharmacokinetics, pregnancy, antiretroviral therapy, HIV seropositivity, infectious disease transmission, vertical, drug monitoring, integrase inhibitors, protease inhibitors, metabolic side effects of drugs and substances

## Abstract

Pregnancy introduces significant physiological changes that alter the pharmacokinetics (PK) of antiretroviral therapy (ART), impacting its safety and efficacy in HIV-positive women. Optimizing ART during pregnancy is critical to maintaining maternal virological suppression and preventing mother-to-child transmission (MTCT) of HIV. This review evaluates the impact of pregnancy-induced PK changes on ART and proposes strategies for tailored regimens to improve outcomes. A comprehensive review of published literature was conducted, focusing on PK adaptations during pregnancy and their implications for different ART classes, including protease inhibitors (PIs), integrase strand transfer inhibitors (INSTIs), and nucleoside reverse transcriptase inhibitors (NRTIs). Key studies were analyzed to assess drug exposure, efficacy, and safety. Pregnancy significantly alters the PK of antiretrovirals, with increased hepatic metabolism, renal clearance, and changes in plasma protein binding leading to reduced drug exposure. For example, drugs like lopinavir and atazanavir require dose adjustments, while dolutegravir maintains efficacy despite reduced plasma levels. Integrase inhibitors demonstrate favorable virological suppression, although cobicistat-boosted regimens show subtherapeutic levels. Tailored approaches, such as therapeutic drug monitoring (TDM), optimize ART efficacy while minimizing toxicity. Pregnancy-specific PK changes necessitate evidence-based ART adjustments to ensure virological suppression and reduce MTCT risk. Incorporating TDM, leveraging pharmacogenomic insights, and prioritizing maternal and neonatal safety are critical for personalized ART management. Further research into long-acting formulations and global guideline harmonization is needed to address disparities in care and improve outcomes for HIV-positive pregnant women.

## 1. Introduction

The management of HIV infection in pregnancy poses unique pharmacological challenges due to the significant physiological changes that occur during this period [1,2]. These changes, including altered drug metabolism, increased plasma volume, and modified enzyme activity, impact the pharmacokinetics (PK) of antiretroviral therapy (ART), necessitating careful dose optimization to ensure efficacy and safety for both the mother and the fetus [3]. The interplay between these physiological adaptations and ART regimens is critical, as inadequate drug exposure can compromise maternal virologic suppression and increase the risk of vertical transmission of HIV [3].

Pregnancy induces notable PK modifications that affect drug absorption, distribution, metabolism, and excretion [1,2]. Specifically, increased activity of hepatic enzymes such as CYP3A4 and UGT1A1, along with decreased plasma protein binding, has been shown to reduce the exposure of key antiretroviral agents during gestation [4]. For instance, integrase strand transfer inhibitors (INSTIs) such as bictegravir and protease inhibitors like darunavir exhibit significantly lower plasma concentrations during the second and third trimesters compared to the postpartum period [5,6]. Despite these reductions, some regimens maintain therapeutic efficacy, as evidenced by their ability to achieve virologic suppression and prevent mother-to-child transmission (MTCT) [7].

Current guidelines recommend the use of ART regimens that balance maternal health with neonatal safety, emphasizing the prevention of MTCT as a primary outcome [8]. Drugs such as bictegravir/emtricitabine/tenofovir alafenamide (B/F/TAF) have demonstrated their utility in maintaining virologic suppression throughout pregnancy, despite reduced PK exposure [5]. These findings underline the importance of monitoring maternal plasma concentrations and adjusting regimens when necessary to sustain antiviral activity. Similarly, the placental transfer of ART drugs, which ensures fetal protection against HIV, remains a critical factor in selecting optimal therapies.

While pregnancy-induced physiological changes are well-documented, their implications for ART dosing strategies remain inadequately addressed. Many current studies lack sufficient population-based data, do not assess long-term neonatal safety, or fail to integrate therapeutic drug monitoring (TDM) recommendations. For example, protease inhibitors such as darunavir and cobicistat-boosted regimens exhibit reduced plasma levels during pregnancy, necessitating dose adjustments and closer monitoring. Integrase inhibitors like dolutegravir, despite their efficacy, require further study to refine dosing strategies, particularly given initial concerns regarding neural tube defects, which have not been consistently confirmed in subsequent research [9,10].

This review addresses the critical gaps in the literature by providing a comprehensive evaluation of pregnancy-induced PK changes and their implications for ART optimization. It examines the limitations of existing studies, including inconsistent dosing recommendations, limited TDM application, and lack of pharmacogenomic integration, while proposing evidence-based ART modifications tailored to pregnancy-specific PK alterations. By addressing these challenges, this review aims to advance the clinical management of HIV in pregnant women, ensuring optimal maternal virologic suppression, fetal protection, and improved ART adherence.

## 2. Physiological Changes During Pregnancy and Their Impact on Drug Pharmacokinetics

This work was conducted as a narrative literature review to summarize and analyze the pharmacokinetic (PK) changes in ART during pregnancy. Given its narrative nature, the literature selection was based on clinical relevance and expert judgment, rather than a strict systematic approach. A PubMed search was performed using the query “pharmacokinetics AND pregnancy AND HIV”, applying filters to include clinical studies published within the last ten years. Additionally, relevant review articles and international guidelines were included based on the expertise of two team members—a professor of obstetrics and gynecology and a professor of pharmacology. We used ChatGPT-4.0 to support the extraction of data from the references.

Unlike systematic reviews, this study did not follow predefined inclusion/exclusion criteria but rather aimed to integrate key findings from diverse sources, prioritizing studies that provided clinically relevant insights into drug class-specific PK alterations, dose adjustments, and therapeutic drug monitoring (TDM). Since this was not a quantitative synthesis, no formal evidence grading was applied; instead, findings were thematically analyzed, focusing on pharmacokinetic trends, clinical implications, and challenges in ART optimization during pregnancy. This approach aimed to highlight knowledge gaps and guide future research in the field.

### 2.1. General Physiological Alterations During Pregnancy

During pregnancy, the respiratory system adapts to meet increased oxygen demands for both the mother and fetus. This includes an increased tidal volume to enhance oxygen intake, while the expanding uterus exerts pressure on the diaphragm, slightly reducing lung capacity and potentially limiting deep breaths. Additionally, respiratory rate increases to ensure adequate oxygen supply. These physiological changes support fetal development and maternal metabolic needs [1,2].

The cardiovascular system of the pregnant woman undergoes a series of fundamental adaptations to support fetal growth and maternal metabolic needs. First, there is a notable increase in blood volume, influenced by the activity of the renin–angiotensin–aldosterone axis, which promotes sodium reabsorption at the renal level. This increase in blood volume is associated with cardiovascular changes such as an increase in cardiac output due to increased plasma volume, greater preload, and consequently a greater ejection volume of the left ventricle. Additionally, the elevation of heart rate and the reduction in peripheral vascular resistance contribute to this increase [1,2].

The reduction in peripheral vascular resistance during pregnancy is driven by hormonal and vascular adaptations. Progesterone and estrogens induce vasodilation by relaxing vascular smooth muscle, increasing nitric oxide production, and reducing vasoconstrictors like endothelin-1. Relaxin directly promotes vasodilation in uteroplacental circulation, while prostaglandins contribute to vascular relaxation. Additionally, vascular remodeling increases vessel diameter and enhances blood flow capacity, particularly in the uterus and placenta. These mechanisms are essential to maintaining adequate placental perfusion and ensuring optimal fetal development [11].

The digestive system of the woman undergoes adaptations to meet the changing demands of the body and the fetus. Pregnancy hormones such as progesterone cause a decrease in peristalsis, which can result in an increase in intestinal transit time. Progesterone causes a decrease in intestinal motility, prolonged gastric emptying, and consequently, an increase in gastric pH, which could reduce the absorption of antiretrovirals (ARVs) and compromise their bioavailability, especially in the case of slow-absorbing medications or those dependent on pH. However, while this prolonged transit may theoretically influence drug absorption, clinical studies suggest that the overall impact on oral drug bioavailability varies depending on factors such as gastric emptying, enzymatic metabolism, and transporter activity [12]. Evidence has shown that the outcome in these pharmacodynamic parameters also depends on additional factors, such as plasma protein binding and drug clearance due to metabolism by isoenzymes like cytochrome P450 (CYP3A), whose expression is increased by progesterone [13]. At the intestinal level, not only is there a prolongation in transit time, but also changes occur in transporter proteins that influence the bioavailability and effectiveness of the drug, which in this case is measured in terms of the reduction in viral load. One example of this is seen in studies evaluating the changes in the effectiveness of HIV integrase inhibitors during pregnancy. For instance, raltegravir absorption increases with the rise in gastric pH, but its stronger binding to P-glycoprotein, whose expression is increased during pregnancy, on the other hand, decreases its absorption. However, studies have shown that despite this, there were no changes in drug exposure or half-life when comparing pregnant and postpartum patients that were significant for clinical practice [7]. The growing uterus exerts pressure on the digestive organs, contributing to symptoms such as heartburn, indigestion, and constipation. Additionally, hormonal fluctuations and changing nutritional needs can affect appetite and food preferences. Nausea and vomiting, common in the first trimester, are associated with hormonal changes and adaptations to pregnancy. To meet the nutritional needs of the fetus, the body may increase the absorption of nutrients such as iron and calcium [14].

The significant increase in blood volume directly impacts the genitourinary system. The kidneys experience an increase in glomerular filtration rate, facilitating the elimination of waste products and maintaining fluid balance. However, the growing uterus can exert pressure on the bladder, causing an increase in urinary frequency. This pressure also affects the ureters and, in some cases, can contribute to renal dilation and increase susceptibility to urinary infections. Hormonal changes such as increased progesterone influence the smooth muscles of the ureters and bladder, impacting urinary function and control [15].

**Figure 1:**
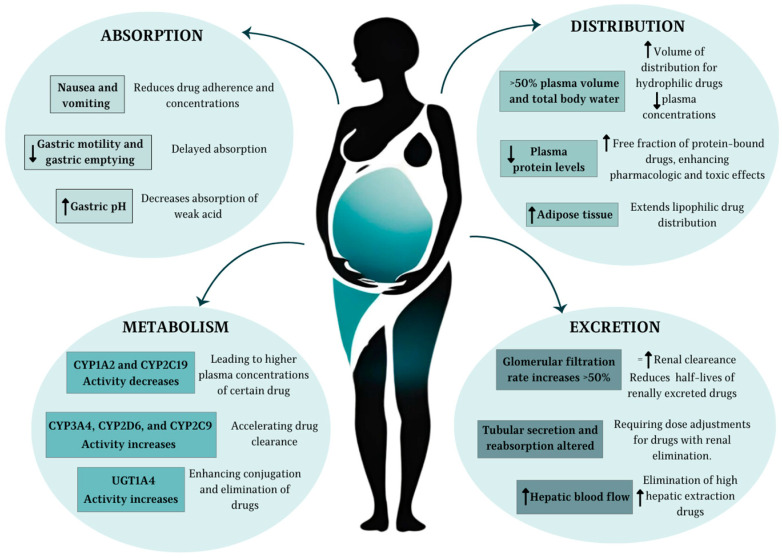
Most relevant pharmacokinetic changes during pregnancy. ↑ indicates an increase, ↓ a decrease, and = no change.

Within the endocrine system, there is a considerable increase in the production of several key hormones, such as the chorionic gonadotropin hormone (HCG) for maintaining early pregnancy, progesterone and estrogen (E2-estradiol) to prepare the uterus and body for gestation, prolactin for future milk production, and growth hormone to support fetal growth. Additionally, thyroid function is affected, with an increase in production of the thyroid hormone. The adrenal glands also undergo changes, increasing the production of cortisol and aldosterone to manage stress and maintain electrolyte balance. There is also greater insulin resistance, ensuring an adequate supply of glucose for the developing fetus [16]. The most important physiological changes in pregnancy, which in turn lead to pharmacokinetic modifications of clinical significance, are shown in Figure 1.

### 2.2. Specific Effects on Absorption, Distribution, Metabolism, and Excretion

#### 2.2.1. Absorption

Drug absorption during pregnancy is influenced by a series of physiological changes in the gastrointestinal (GI) system. Reduced gastric acid secretion and increased gastric pH, driven by hormonal changes such as elevated progesterone, can alter the solubility and ionization of drugs. Weak acids (e.g., aspirin) tend to ionize more at higher pH levels, which may reduce their absorption, while weak bases (e.g., caffeine) remain largely unionized, potentially enhancing their bioavailability. Delayed gastric emptying and slower intestinal transit time, also attributed to progesterone, can modify the time to peak drug concentration (Tmax), particularly for orally administered drugs. These changes may have a greater impact on medications requiring the rapid onset of action [12].

Nausea and vomiting, particularly during the first trimester, may significantly impair oral drug absorption, emphasizing the need for timing medication intake to periods when symptoms are less pronounced. Additionally, increased intestinal blood flow and vasodilation during pregnancy may facilitate drug absorption for certain formulations. Despite these physiological alterations, the overall bioavailability of most drugs remains relatively unaffected with repeated dosing [12]. However, limited data exist on the impact of these changes on other routes of drug administration, such as intramuscular or transdermal delivery [1,2].

#### 2.2.2. Distribution

The distribution of drugs is profoundly affected by the increased plasma volume (up to 50%) and total body water (approximately 8 L) that occur during pregnancy. These changes result in an expanded volume of distribution (Vd) for hydrophilic drugs, leading to reduced plasma concentrations and, potentially, diminished therapeutic effects. For lipophilic drugs, the increase in maternal adipose tissue, which serves as an additional reservoir, further extends their distribution and may delay the achievement of steady-state levels [1,2].

Another critical factor is the reduction in plasma protein concentrations, particularly albumin and alpha-1-acid glycoprotein. This leads to a higher free fraction of protein-bound drugs, potentially enhancing their pharmacologic and toxic effects. For example, a decrease in albumin binding from 99% to 98% for a highly protein-bound drug effectively doubles the active free drug concentration. This phenomenon is clinically significant for medications with narrow therapeutic windows or those requiring precise plasma concentration monitoring [1,2].

Antiretroviral drugs exhibit varying degrees of plasma protein binding, which influences their pharmacokinetics during pregnancy. Protease inhibitors (PIs) such as darunavir and integrase strand transfer inhibitors (INSTIs) like bictegravir are highly protein-bound (~95–99%), while nucleoside reverse transcriptase inhibitors (NRTIs) such as tenofovir and lamivudine have lower binding (<50%). The reduction in plasma proteins during pregnancy may increase the free fraction of these drugs, but it also facilitates faster metabolism and clearance, contributing to decreased total plasma concentrations. Enhanced uteroplacental perfusion and the addition of the feto-placental compartment further redistribute these drugs, complicating pharmacokinetic predictions [7]. Consequently, while free drug concentrations may transiently increase, overall drug exposure often declines, necessitating careful monitoring to ensure therapeutic efficacy and prevent mother-to-child transmission (MTCT).

Regarding this matter, the evidence supports the fact that plasma protein binding for antiretroviral drugs is highly variable. For example, for the case of raltegravir, the latest evidence shows that despite a decrease in serum albumin concentrations during pregnancy by 46% and known affinity of raltegravir, the predicted unbound fraction was decreased by <1%. Based on this statement, raltegravir is not relevantly impacted by pregnancy and changes in total plasma concentrations are unlikely to be the result of changed protein binding [7].

An open label, multicenter, single-arm, phase 1b study conducted in 33 virologically suppressed pregnant women with HIV-1 showed that exposure of bictegravir/emtricitabine/tenofovir alafenamide were lower during pregnancy than postpartum [5]. However, the mean Bictegravir concentration was maintained at levels that indicated efficacious exposure through adequate virologic suppression, meaning no dose adjustment was required during pregnancy, despite changes in pharmacodynamics, such as that of low protein binding [5], which means that even though a change in this parameter has been observed, there is no clinical impact on dose adjustment for this specific drug in pregnant women.

#### 2.2.3. Metabolism

Hepatic drug metabolism undergoes significant modulation during pregnancy due to hormonal changes, including elevated levels of estrogen and progesterone. Phase I metabolism, primarily mediated by cytochrome P450 enzymes, is notably altered. Enzymes such as CYP3A4, CYP2D6, and CYP2C9 exhibit increased activity, leading to enhanced clearance of their substrates, including certain antiretrovirals like protease inhibitors. Conversely, CYP1A2 and CYP2C19 show reduced activity, potentially resulting in higher plasma concentrations of substrates metabolized by these pathways [17].

Table 1 presents a summary of the role of cytochrome P450 (CYP) in the metabolism of the most important antiretroviral drugs.

Phase II metabolism, involving conjugation reactions such as glucuronidation and sulfation, also experiences changes. For instance, uridine diphosphate glucuronosyltransferase (UGT) activity increases significantly, particularly UGT1A4, which contributes to the accelerated clearance of drugs like lamotrigine. The interplay between genetic polymorphisms and enzyme activity adds another layer of complexity, as maternal genotype can influence the extent of metabolic alterations. These changes necessitate close monitoring and dose adjustments, particularly for drugs with narrow therapeutic indices [1,2,17].

#### 2.2.4. Excretion

Renal drug excretion is markedly enhanced during pregnancy, driven by a 50% increase in renal blood flow and glomerular filtration rate (GFR), beginning as early as the first trimester and peaking in the third trimester. This leads to increased clearance and reduced half-lives for drugs primarily eliminated via the kidneys, such as cefazolin, digoxin, and lithium. For example, lithium clearance doubles in the third trimester compared to preconception levels, requiring careful dose titration to maintain therapeutic concentrations [19].

Renal tubular secretion and reabsorption also undergo changes, although their impact varies depending on the drug. For instance, while GFR increases uniformly, tubular handling may be drug-specific, affecting renally cleared medications differently. Additionally, the increased renal elimination of hydrophilic drugs underscores the need for higher or more frequent dosing to achieve desired plasma concentrations. These changes, coupled with alterations in maternal sodium and water homeostasis, contribute to a complex renal pharmacokinetic profile during pregnancy [20].

Despite the renal excretion of NRTIs and the physiological changes in renal function during pregnancy, studies indicate that these alterations do not result in clinically significant pharmacokinetic changes, eliminating the need for routine dose adjustments [21]. However, given the observed increase in renal clearance, pharmacokinetic monitoring may be warranted in specific cases to ensure therapeutic efficacy [4]. Similarly, while protease inhibitors (PIs) and non-nucleoside reverse transcriptase inhibitors (NNRTIs) undergo primarily hepatic metabolism, integrase strand transfer inhibitors (INSTIs) such as dolutegravir and raltegravir exhibit partial renal clearance [7]. This suggests that pregnancy-related pharmacokinetic alterations could influence their plasma concentrations, though the clinical significance varies depending on each drug’s primary metabolic pathway.

## 3. Pharmacokinetics of Antiretroviral Drugs in Pregnant Women

Pregnancy alters the PK of ARVs in significant ways, driven by physiological changes such as increased metabolism, enhanced renal clearance, and changes in protein binding. This chapter explores the specific PK adaptations observed in different classes of ARVs, highlighting their implications for efficacy and safety during pregnancy [7,21]. A summary of the most important studies evaluating the pharmacokinetic changes in ARV drugs during pregnancy is presented in Supplementary Materials.

### 3.1. Integrase Strand Transfer Inhibitors (INSTIs)

Dolutegravir (DTG): Studies have shown that dolutegravir’s exposure decreases during pregnancy, primarily due to physiological changes such as increased hepatic enzyme activity and plasma volume. For instance, Mulligan et al. reported reductions in area under the curve (AUC) by 29% in the third trimester and 37% in the second trimester compared to postpartum levels [22]. Despite these reductions, trough concentrations remained above the effective concentration (EC90), ensuring virological suppression and preventing mother-to-child transmission (MTCT). The drug also exhibits efficient placental transfer, with a cord-to-maternal blood ratio of 1.25, suggesting adequate fetal exposure. Additionally, dolutegravir’s high barrier to resistance and its tolerability profile make it an important option for pregnant women. Clinical data further indicate no significant increase in adverse birth outcomes with its use, reinforcing its safety [22].

The pharmacokinetic profile of dolutegravir highlights the balance between maternal therapeutic efficacy and fetal drug exposure. As part of combination antiretroviral therapy (cART), DTG demonstrates robust virological suppression even in cases of reduced exposure. Studies emphasize that standard dosing remains adequate during pregnancy without requiring dose adjustments, simplifying its clinical use. However, more extensive population-based studies are warranted to validate its long-term neonatal safety, given its relatively recent inclusion in ART guidelines for pregnant women [21].

Bictegravir (BIC): Zhang et al. found that bictegravir’s plasma concentrations were significantly lower during pregnancy due to increased metabolism via CYP3A4 and UGT1A1. However, these levels were still sufficient to maintain virological suppression, underscoring its potential as a reliable component of ART regimens for pregnant women. Its favorable pharmacokinetic profile and low risk of resistance make it a valuable option for treatment during pregnancy, although ongoing studies are needed to confirm its safety across diverse populations [5].

Elvitegravir/Cobicistat (EVG/COBI): Elvitegravir exposure is markedly reduced during pregnancy, with trough levels often falling below therapeutic thresholds [23]. This reduction is compounded by cobicistat’s inability to maintain adequate boosting during pregnancy, leading to recommendations against its use in this population [24]. The pronounced reduction in EVG/COBI exposure highlights the necessity of using alternative agents to ensure virological suppression and prevent MTCT.

Raltegravir (RAL): Raltegravir’s pharmacokinetics (PK) during pregnancy exhibit high interindividual variability, with studies, including those by Watts et al., reporting a 50% reduction in AUC compared to postpartum levels [25]. Due to decreased plasma concentrations, twice-daily dosing is recommended during pregnancy to ensure therapeutic efficacy. Despite these pharmacokinetic alterations, virological suppression has been maintained in most cases, and raltegravir’s effective placental transfer (cord-to-maternal ratio of 1.5) supports its use in preventing mother-to-child transmission (MTCT) [25]. However, as a first-generation integrase strand transfer inhibitor (INSTI), raltegravir is associated with several resistance-conferring mutations and demonstrates cross-resistance with other INSTIs, such as elvitegravir [21]. In contrast, dolutegravir, a second-generation INSTI, maintains appropriate target levels with standard dosing during pregnancy, further supporting its preferred use in this population [21].

Regarding clinical use, dolutegravir, bictegravir, and raltegravir are the only INSTIs advised for use during pregnancy, with dolutegravir being the preferred option due to its once-daily administration, greater resistance barrier, and more extensive safety data across pregnancy [21].

### 3.2. Protease Inhibitors (PIs)

Darunavir/Ritonavir (DRV/r): Colbers et al. demonstrated that, when administered once daily during pregnancy, darunavir exposure was reduced by 22–34%, though unbound (active) drug levels remained adequate. Twice-daily dosing is preferred to ensure therapeutic trough levels. Placental transfer is minimal, reducing fetal exposure, and no MTCT cases were observed in their cohort. The need for increased dosing in certain cases highlights the importance of therapeutic drug monitoring (TDM) to optimize outcomes [26].

Atazanavir/Ritonavir (ATV/r): Similarly to darunavir, atazanavir plasma concentrations decrease during pregnancy. Studies report up to a 34% reduction in AUC, though levels generally remain above therapeutic thresholds. Despite these changes, the regimen effectively maintains virological suppression and prevents MTCT [27].

Lopinavir/Ritonavir (LPV/r): During pregnancy, significant pharmacokinetic alterations in lopinavir/ritonavir exposure have been observed. Population pharmacokinetic analysis estimated a 17% increase in lopinavir clearance in pregnant women compared to nonpregnant subjects, with clearance values postpartum being 26.4% and 37.1% lower than in nonpregnant and pregnant individuals, respectively [28]. Additionally, pregnancy was associated with a 68% increase in lopinavir/ritonavir clearance, leading to an overall reduction in drug exposure (lopinavir −33%, ritonavir −17%) compared to well-nourished controls, likely due to decreased bioavailability. Food insecurity further exacerbated these reductions, with significant pharmacokinetic variability explained by hair concentrations [29]. Although a standard dose of 400/100 mg twice daily has shown similar efficacy in pregnant and nonpregnant women, increasing the dose to 600/150 mg twice daily during the third trimester results in lopinavir exposure levels comparable to those observed in nonpregnant adults receiving standard dosing [30]. Consequently, dose adjustments should be considered during pregnancy to maintain adequate drug exposure, while postpartum dosing can be reduced to standard levels before two weeks postpartum.

Protease inhibitors (PIs) undergo significant pharmacokinetic alterations during pregnancy due to physiological changes and potential genetic factors affecting CYP3A4 metabolism. PIs exhibit extensive drug interactions and are associated with an increased risk of adverse pregnancy outcomes, including preterm birth and small-for-gestational-age infants, likely due to hormonal and placental effects. Despite these concerns, PIs demonstrate similar virologic efficacy and perinatal HIV transmission rates compared to other antiretroviral classes. Given their pharmacokinetic challenges, potential adverse effects, and the availability of alternative regimens, PIs are less frequently recommended as first-line therapy in high-resource settings [21].

### 3.3. Non-Nucleoside Reverse Transcriptase Inhibitors (NNRTIs)

Rilpivirine (RPV): Rilpivirine pharmacokinetics are influenced by pregnancy-related physiological changes, including alterations in cardiac output, protein binding, volume of distribution, and CYP3A4 activity. In the IMPAACT P1026s study, rilpivirine exposure (AUC0–24 and C24) was significantly lower during the second and third trimesters compared to postpartum (*p* < 0.05), with high interindividual variability. The median cord blood/maternal concentration ratio was 0.55. Virologic suppression (HIV-1 RNA ≤50 copies/mL) was achieved in 70% of women at delivery. Minimum rilpivirine concentrations were significantly lower in visits with detectable viremia (*p* = 0.0004), though 90% of participants maintained concentrations above the protein-binding adjusted EC90. These findings indicate reduced rilpivirine exposure during pregnancy, warranting careful monitoring to ensure virologic efficacy [31].

Etravirine (ETR): Etravirine pharmacokinetics were evaluated in a phase IIIb open-label study of HIV-1-infected pregnant women receiving 200 mg twice daily as part of combination antiretroviral therapy. Among 13 participants with available pharmacokinetic profiles, total etravirine exposure was 1.2- to 1.4-fold higher during pregnancy compared to postpartum, though differences were less pronounced for unbound etravirine. Virologic response was maintained, with no cases of perinatal HIV transmission [32].

Non-nucleoside reverse transcriptase inhibitors (NNRTIs) are metabolized primarily via the CYP 450 system, leading to altered plasma concentrations during pregnancy. While efavirenz and rilpivirine levels decrease, etravirine levels increase, and nevirapine remains unchanged. Despite these pharmacokinetic variations, dose adjustments are not recommended, though closer viral load monitoring may be beneficial. NNRTIs have high placental transfer and effectively prevent vertical HIV transmission but are generally reserved for second- or third-line therapy due to their high susceptibility to resistance and lower efficacy compared to other antiretroviral classes. First-generation NNRTIs (nevirapine and efavirenz) have a lower resistance threshold, while second-generation NNRTIs (etravirine, rilpivirine, doravirine) demonstrate improved resistance profiles. Concerns regarding fetal safety remain, particularly with efavirenz, which has been linked to neurodevelopmental issues, and nevirapine, which has been associated with adverse birth outcomes. NNRTIs may also increase the risk of gestational diabetes, hypertensive disorders, and hepatotoxicity, particularly in pregnancy. Given these considerations, NNRTIs are not recommended as first-line therapy in pregnancy due to resistance concerns, potential adverse birth outcomes, and limited safety data [21].

### 3.4. Nucleoside/Nucleotide Reverse Transcriptase Inhibitors (NRTIs)

Tenofovir: Intracellular tenofovir diphosphate (TFV-DP) concentrations in dried blood spots (DBSs) were evaluated in pregnant and postpartum adolescent girls and young women (AGYW) receiving directly observed pre-exposure prophylaxis (PrEP) in sub-Saharan Africa. TFV-DP half-life was shorter in pregnancy (median 10 days) than postpartum (median 17 days), with steady-state concentrations achieved by weeks 5 and 8, respectively. Median steady-state TFV-DP levels were significantly lower in pregnancy (965 fmol/punch) compared to postpartum (1406 fmol/punch, *p* = 0.006). Population pharmacokinetic modeling confirmed these findings, showing approximately one-third lower TFV-DP concentrations during pregnancy. Baseline creatinine clearance was associated with TFV-DP levels [33].

Emtricitabine (FTC): The study by Hirt et al. assessed emtricitabine (FTC) pharmacokinetics in pregnant women and neonates to determine the optimal neonatal prophylactic dose for preventing mother-to-child HIV transmission. HIV-infected pregnant women received a 400 mg FTC dose at labor initiation, followed by 200 mg daily for seven days postpartum. FTC exhibited good placental transfer (80%), with median maternal and cord blood concentrations at delivery of 1.16 mg/L and 0.72 mg/L, respectively. Pharmacokinetic modeling indicated that a neonatal dose of 1 mg/kg immediately after birth or 2 mg/kg at 12 h postpartum would achieve plasma concentrations comparable to adult values. These findings support tailored neonatal dosing to optimize prophylactic efficacy [34].

Lamivudine (3TC): The study by Benaboud et al. characterized lamivudine (3TC) pharmacokinetics in HIV-infected pregnant and nonpregnant women, as well as fetuses, using population modeling. Lamivudine followed a two-compartment model with linear absorption and elimination. Pregnant women exhibited a 22% higher apparent clearance than nonpregnant women, but this increase did not result in subtherapeutic exposure, suggesting no need for dose adjustment. Placental transfer was substantial, with a fetal-to-maternal AUC ratio of 0.86, and lamivudine accumulated in amniotic fluid (amniotic fluid-to-fetal AUC ratio: 2.9) [35].

NRTIs remain the backbone of antiretroviral therapy in pregnancy due to their efficacy, high placental transfer, and favorable safety profile. Despite increased renal clearance, pharmacokinetic studies indicate no need for dose adjustments, though genetic variability may influence individual drug levels. NRTIs have fewer drug interactions than other ARV classes but require monitoring with rifampin and carbamazepine. Resistance, particularly M184V, remains a concern, with reported rates up to 15% in postpartum women. No significant increase in birth defects has been observed, and while historical data suggested adverse obstetric outcomes, recent studies show mixed results. TDF/FTC is preferred for its association with higher neonatal birth weight and reduced placental oxidative stress, whereas TAF shows better maternal renal safety. Older NRTIs, such as zidovudine and stavudine, are linked to myopathy, neuropathy, and lactic acidosis, reinforcing the preference for TDF/FTC- or ABC/3TC-based regimens [21].

### 3.5. Drug Interactions in ART Regimens

ART and Tuberculosis (TB) Treatments: The study by Gausi et al. examined pregnancy-related and genetic factors affecting isoniazid and efavirenz pharmacokinetics in 847 women receiving isoniazid for 28 weeks (during pregnancy or postpartum), including 786 women on efavirenz. After adjusting for *NAT2* and *CYP2B6* genotypes and weight, pregnancy increased isoniazid clearance by 26% and efavirenz clearance by 15%, leading to reduced drug exposure. Additionally, isoniazid decreased efavirenz clearance by 7% in normal CYP2B6 metabolizers and 13% in slow/intermediate metabolizers. The main determinants of drug concentration were *NAT2* and *CYP2B6* genotypes, which caused a five-fold variation in drug levels between rapid and slow metabolizers [36].

ART and Malaria Treatments: Studies on the pharmacokinetic interactions between efavirenz-based ART and antimalarial drugs in pregnant women indicate significant reductions in drug exposure, potentially impacting treatment efficacy. Dihydroartemisinin-piperaquine (DHA-PQ) exposure was significantly lower in pregnancy, with further reductions in HIV-infected women on efavirenz, suggesting the need for dose modifications. However, increased unbound piperaquine in efavirenz-treated women may partially compensate for reduced total drug levels, warranting further study [37,38]. Artemether-lumefantrine (AL) exposure was also reduced in pregnant women receiving efavirenz, with artemether and DHA concentrations decreasing by up to 76% and 46%, respectively, and lumefantrine exposure lowered by 61–81% on days 7 and 14 [39]. A separate study found that while efavirenz reduces lumefantrine exposure in nonpregnant adults, pregnancy attenuates this effect, leading to higher lumefantrine levels in pregnant women [40]. These findings highlight the need for careful dose optimization of DHA-PQ and AL in HIV-infected pregnant women on efavirenz to ensure effective malaria treatment and prevention.

The pharmacokinetic changes during pregnancy necessitate careful selection and monitoring of antiretroviral regimens. While most ARVs maintain efficacy with standard dosing, specific drugs, such as elvitegravir/cobicistat, require avoidance or adjustments. Understanding these PK adaptations ensures optimal maternal and fetal outcomes, supporting the global goal of eliminating mother-to-child transmission (MTCT).

The pharmacokinetic alterations of antiretroviral therapy (ART) during pregnancy necessitate careful drug selection to ensure both maternal virologic suppression and fetal safety. Dolutegravir remains the preferred integrase inhibitor due to its once-daily dosing, high resistance barrier, and adequate drug levels despite reduced exposure. Bictegravir is considered an option during pregnancy, whereas elvitegravir/cobicistat exhibits significantly decreased concentrations, compromising its efficacy. Raltegravir remains an option with twice-daily dosing to maintain therapeutic levels. Among protease inhibitors, ritonavir-boosted regimens require dose adjustments, particularly for lopinavir/ritonavir, which should be increased to 600/150 mg twice daily in the third trimester. Non-nucleoside reverse transcriptase inhibitors (NNRTIs) exhibit variable pharmacokinetics, with reduced levels of efavirenz and rilpivirine but increased exposure to etravirine. While NNRTIs are not first-line due to resistance concerns and potential adverse pregnancy outcomes, they remain viable alternatives. Nucleoside reverse transcriptase inhibitors (NRTIs) form the backbone of ART in pregnancy, with tenofovir/emtricitabine and abacavir/lamivudine being preferred due to their safety profiles. Tenofovir alafenamide offers improved renal safety compared to tenofovir disoproxil fumarate. Drug interactions further complicate ART management, particularly with tuberculosis and malaria treatments. Isoniazid reduces efavirenz exposure, with genetic variations in *NAT2* and *CYP2B6* playing a key role in drug metabolism. Efavirenz also reduces exposure to dihydroartemisinin-piperaquine and artemether-lumefantrine, potentially necessitating dose modifications for effective malaria treatment. These findings underscore the need for close monitoring and, in certain cases, dose adjustments to maintain therapeutic efficacy and prevent mother-to-child transmission (MTCT). Optimizing ART regimens based on pregnancy-specific pharmacokinetics is essential for achieving favorable maternal and neonatal outcomes.

## 4. Efficacy and Safety of ART Regimens During Pregnancy

The efficacy and safety of ART regimens during pregnancy are paramount for achieving virological suppression, preventing MTCT, and ensuring maternal and neonatal health. Pregnancy-specific PK changes challenge the consistency of ART efficacy, necessitating robust clinical evidence to inform regimen selection and dose adjustments. This chapter systematically examines the evidence supporting ART efficacy and safety in pregnancy, focusing on virological suppression rates, MTCT outcomes, and maternal and fetal safety.

### 4.1. Virological Suppression During Pregnancy

Maintaining virological suppression (HIV RNA < 50 copies/mL) is critical for preventing MTCT of HIV. Clinical studies consistently report high suppression rates with appropriately tailored ART regimens. For example, Mulligan et al. demonstrated that despite a 37% reduction in dolutegravir (DTG) exposure during the second trimester, 98% of pregnant women achieved virological suppression at delivery [22]. This highlights the resilience of INSTI-based regimens in maintaining efficacy despite PK challenges.

Similarly, atazanavir/ritonavir (ATV/r)-based regimens have demonstrated robust suppression rates. In a cohort study, Colbers et al. reported that 81% of women treated with ATV/r achieved undetectable viral loads at delivery, highlighting its efficacy despite reduced drug exposure during pregnancy [27].

Despite the pharmacokinetic changes observed in ART during pregnancy, including reduced drug exposure for certain regimens, most ART combinations maintain sufficient virological suppression. Protease inhibitor-based regimens such as ATV/r and lopinavir/ritonavir (LPV/r) have demonstrated effective viral load suppression at delivery, with studies reporting over 80% of pregnant women achieving HIV RNA levels below 50 copies/mL. The findings also emphasize that maintaining adherence to ART and regular monitoring of viral load are essential to optimizing treatment outcomes and preventing perinatal HIV transmission [21].

### 4.2. Prevention of Mother-to-Child Transmission (MTCT)

Effective prevention of MTCT of HIV depends on achieving and maintaining maternal virological suppression through optimized ART. Without intervention, MTCT rates range from 15% to 40%, but with combination ART (cART), this risk decreases to below 2% [27].

Current guidelines, including those from the U.S. Department of Health and Human Services (DHHS), recommend integrase strand transfer inhibitors (INSTIs) and ritonavir-boosted protease inhibitors (PIs) as preferred regimens during pregnancy due to their high efficacy and established safety profiles [41]. Among INSTIs, dolutegravir (DTG) has demonstrated high virological suppression rates (98%) at delivery, making it a preferred option for pregnant individuals. Clinical trials confirm its superior efficacy compared to efavirenz-based regimens, with fewer adverse pregnancy outcomes when combined with tenofovir alafenamide fumarate (TAF) [42].

Protease inhibitor-based regimens also play an important role in MTCT prevention. Atazanavir/ritonavir (ATV/r) regimens have demonstrated effective viral suppression in over 80% of pregnant women at delivery, reinforcing their clinical utility [27]. However, certain protease inhibitors, such as lopinavir/ritonavir (LPV/r), require dose adjustments during the third trimester to maintain therapeutic levels, given the increased drug clearance observed during pregnancy [43].

Nucleoside reverse transcriptase inhibitors (NRTIs), particularly tenofovir disoproxil fumarate (TDF) and lamivudine (3TC), remain essential components of ART regimens. Although pregnancy alters renal function, studies show that TDF exposure decreases by approximately one-third during pregnancy, but dose adjustments are not required [44,45]. Maintaining adherence to ART throughout pregnancy and postpartum is critical, as suboptimal adherence significantly increases the risk of viral rebound and perinatal HIV transmission [41].

Given the evolving landscape of ART and the emergence of newer regimens, continuous pharmacovigilance and adherence to evidence-based guidelines remain essential for further reducing MTCT rates globally [46].

### 4.3. Maternal Safety and Tolerability

Antiretroviral therapy (ART) during pregnancy must balance maternal safety, viral suppression, and fetal well-being. Integrase strand transfer inhibitors (INSTIs) are generally well-tolerated, with dolutegravir being preferred due to its high resistance barrier and favorable pharmacokinetics, though concerns about hypertensive disorders remain inconclusive. Non-nucleoside reverse transcriptase inhibitors (NNRTIs) carry a higher risk of hepatotoxicity and skin reactions, with efavirenz specifically linked to neuropsychiatric effects and a potential increase in gestational diabetes. Protease inhibitors (PIs), while historically used in pregnancy, have been associated with adverse outcomes such as preterm birth and placental abnormalities [21]. Atazanavir, in particular, may lead to maternal hyperbilirubinemia, potentially resulting in neonatal hyperbilirubinemia [47].

Newer ART classes, such as entry inhibitors and capsid inhibitors, have limited pregnancy safety data. Maraviroc has been linked to hepatotoxicity, while ibalizumab may cause transient neonatal immunosuppression. Lenacapavir, a novel capsid inhibitor, is not currently recommended due to insufficient human data. Given these considerations, ART regimens should prioritize viral suppression while minimizing maternal adverse effects. Pregnant individuals who are already on a stable regimen with effective viral control should continue their treatment unless significant safety concerns arise. Careful monitoring and individualized management remain essential to optimize both maternal and fetal outcomes [21].

### 4.4. Neonatal Outcomes and Safety

Neonatal outcomes, including birth weight, preterm delivery, and congenital anomalies, are critical metrics for evaluating ART safety. Studies indicate no significant increase in adverse neonatal outcomes with most ART regimens. For example, DTG exposure during pregnancy has not been associated with neural tube defects, alleviating earlier concerns raised by preclinical data [10]. Furthermore, regimens containing emtricitabine (FTC) and tenofovir disoproxil fumarate (TDF) have demonstrated favorable safety profiles, with minimal impact on fetal growth and bone mineralization [48].

PIs, despite their metabolic side effects, have shown reassuring neonatal outcomes. Large cohort studies, including the French Perinatal Cohort and PHACS, found no association between lopinavir/ritonavir (LPV/r) exposure and congenital anomalies, with a congenital abnormality rate (2.9%) comparable to HIV-unexposed populations [49]. The Antiretroviral Pregnancy Registry also reported no increased risk of birth defects, including cardiovascular and genitourinary anomalies, with a prevalence of 2.1% (95% CI, 1.4–2.9%), like the general U.S. population [50].

However, LPV/r has been linked to increased preterm birth and low birth weight. The PROMISE study reported higher rates of low birth weight with LPV/r-based regimens [51], while multiple studies, including PHACS SMARTT, a Chinese cohort, and UK/Ireland data, found an elevated risk of preterm delivery compared to NNRTI-based regimens [41]. These findings underscore the need to balance maternal virologic control with potential neonatal risks when selecting ART in pregnancy.

However, while these findings are reassuring, certain limitations should be acknowledged. Many studies evaluating neonatal outcomes are observational and may be subject to selection bias, as women receiving ART often differ in baseline characteristics such as nutritional status, comorbidities, and access to prenatal care, which could influence neonatal health independently of ART exposure. Additionally, the heterogeneity of study designs, variations in ART regimens used, and differences in population demographics may limit the generalizability of these results. Furthermore, most studies focus on short-term neonatal outcomes, while longitudinal research is still needed to assess potential long-term effects of in utero ART exposure, particularly regarding neurodevelopment, immune function, and metabolic health. Future studies should aim for standardized methodologies, larger cohorts, and extended follow-up periods to provide robust evidence on the long-term safety of ART during pregnancy.

## 5. Therapeutic Drug Monitoring (TDM) and Dose Adjustments in Pregnancy

The TDM of ART in pregnancy plays a crucial role in ensuring effective maternal viral suppression and minimizing the risk of MTCT of HIV. Pregnancy induces significant PK alterations, including changes in drug metabolism, absorption, and clearance, which may lead to subtherapeutic drug levels and potential virologic failure. Historically, PIs were the preferred third agents in triple ART due to their resistance barrier, but reduced plasma concentrations during pregnancy increased concerns about viral escape and MTCT. In contrast, INSTIs have gained preference due to their rapid viral load reduction, favorable safety profile, and lack of need for pharmacokinetic boosting. Despite early concerns regarding neural tube defects with DTG use in pregnancy, recent data from Botswana and Brazil indicate that the risk is lower than previously thought, prompting international guidelines to recommend INSTIs as first-line therapy [7,52].

The role of TDM in pregnancy has shifted towards monitoring ART effectiveness in cases of virologic failure, drug–drug interactions, and continued use of pharmacokinetically affected drugs like elvitegravir/cobicistat (EVG/c). Studies indicate that subtherapeutic antiretroviral drug levels in pregnancy correlate with an increased risk of virologic failure postpartum, emphasizing the importance of longitudinal monitoring. While TDM has proven valuable for NNRTIs and PIs, its applicability remains limited for NRTIs due to their intracellular phosphorylation requirements. Despite challenges such as cost, limited access to assays, and variability in adherence assessment, TDM remains a critical tool in ensuring optimal ART exposure during pregnancy, reducing MTCT, and improving maternal and neonatal health outcomes [52].

The optimal sampling point for evaluating ART efficacy in pregnancy is crucial to ensuring therapeutic drug levels. Trough concentrations (C_trough), measured just before the next scheduled dose, are the most reliable indicators of whether drug levels remain within the therapeutic range throughout the dosing interval. This is particularly relevant for ARVs with short half-lives, significant pharmacokinetic changes during pregnancy, or a narrow therapeutic window [53].

In conclusion, TDM plays a pivotal role in optimizing ART during pregnancy by ensuring adequate drug exposure, preventing MTCT, and minimizing maternal toxicity. However, its accessibility remains limited in many settings, such as in Colombia, highlighting the need for equitable access to this resource. Overcoming these challenges and leveraging technological advancements will enhance TDM integration into pregnancy-specific ART management, ultimately improving maternal and neonatal outcomes.

## 6. Implications for Clinical Practice and Guideline Development

The pharmacokinetic (PK) adaptations during pregnancy significantly impact antiretroviral therapy (ART) efficacy, necessitating evidence-based clinical practices and guidelines to optimize maternal and neonatal outcomes. Translating PK insights into clinical practice requires tailored ART regimen selection, dose adjustments, and therapeutic drug monitoring (TDM) to prevent virological failure and reduce the MTCT. Reduced plasma concentrations observed with PIs such as lopinavir/ritonavir and atazanavir/ritonavir necessitate either dose adjustments or more frequent monitoring. Certain experts suggest higher doses of lopinavir/ritonavir (LPV/r) during the second and third trimesters of pregnancy, recommending LPV/r 600/150 mg or 500/125 mg twice daily, particularly for women with prior protease inhibitor (PI) exposure or those initiating treatment during pregnancy with a baseline viral load exceeding 50 copies/mL. However, once-daily dosing of LPV/r is not advised during pregnancy [41]. During the second and third trimesters of pregnancy, if atazanavir/ritonavir is administered alongside tenofovir-DF or an H2-receptor antagonist, it is recommended to increase the dose to atazanavir/ritonavir 400/100 mg to maintain therapeutic drug levels [54].

Similarly, integrase strand transfer inhibitors (INSTIs), particularly dolutegravir, have become a preferred option due to their high resistance barrier and favorable safety profile [7,21]. Despite reductions in plasma concentrations during pregnancy, dolutegravir maintains therapeutic trough levels in most cases, although close monitoring is recommended in patients receiving enzyme inducers such as rifampicin. In contrast, elvitegravir/cobicistat should be avoided due to significant pharmacokinetic reductions that lead to subtherapeutic levels [23].

Nucleoside reverse transcriptase inhibitors (NRTIs), such as tenofovir disoproxil fumarate (TDF) and emtricitabine, remain integral to combination ART regimens, but enhanced renal clearance during pregnancy reduces tenofovir levels. Although standard doses of TDF (300 mg once daily) are generally sufficient for virological suppression, therapeutic drug monitoring is recommended in cases of renal impairment to prevent potential renal toxicity [41]. When tenofovir is coadministered with atazanavir, additional monitoring is necessary due to a further decrease in atazanavir exposure. To ensure adequate drug levels, an increased dose of atazanavir/ritonavir (400 mg/100 mg) may be required [55]. Cobicistat-boosted regimens pose another challenge during pregnancy, as cobicistat loses its boosting effect, leading to insufficient drug exposure for protease inhibitors and integrase inhibitors. Consequently, regimens containing elvitegravir/cobicistat or atazanavir/cobicistat should be avoided due to the risk of virological failure [41].

The integration of TDM into routine clinical practice offers a practical approach to optimizing ART regimens. TDM is particularly important for protease inhibitors such as lopinavir and atazanavir, ensuring plasma concentrations remain within therapeutic ranges. It is also useful for drugs with significant pharmacokinetic variability, such as TDF, particularly in individuals with renal dysfunction. Furthermore, TDM is beneficial in patients using enzyme inducers that alter ART metabolism, such as rifampicin. Establishing standardized protocols for sample timing, result interpretation, and dose adjustments will be essential to ensure consistency in clinical decision-making [52,53].

One of the major challenges in implementing pharmacokinetically guided ART adjustments is the resource constraints prevalent in low- and middle-income countries (LMICs). Many LMICs lack the infrastructure for TDM or advanced pharmacokinetic modeling, limiting access to optimized ART for pregnant women. Cost-effective strategies, such as point-of-care TDM technologies, hold promise for addressing these gaps. The development of these tools, alongside standardized training programs for healthcare providers, would facilitate the consistent application of pharmacokinetically optimized ART even in resource-limited settings. Another key consideration is the role of pharmacogenomics in ART metabolism. Genetic polymorphisms, particularly *CYP3A5* variations, significantly influence ART metabolism and efficacy, especially in genetically diverse populations. Future guidelines should incorporate pharmacogenomic testing as part of routine care to tailor ART regimens for individuals with specific metabolic profiles [56].

Ensuring optimal ART during pregnancy requires a focus not only on maternal viral suppression but also on the long-term implications of in utero ART exposure for neonatal health and development. Longitudinal studies are crucial to evaluating potential neurodevelopmental effects, growth parameters, and immune function in infants exposed to ART during gestation. Comparative studies between different ART regimens will provide robust evidence to guide clinical decision-making and address current knowledge gaps regarding safety and efficacy. Emerging therapeutic options, such as long-acting injectable formulations, hold promise for improving adherence and maintaining consistent drug levels, particularly in populations with limited healthcare access. However, their use during pregnancy remains uncertain, as pharmacokinetic variability—such as the significant reduction in rilpivirine concentrations—may compromise efficacy [57]. Therefore, rigorous evaluation in pregnant populations is essential before these formulations can be integrated into standard clinical practice.

The widespread implementation of antiretroviral therapy (ART) in pregnancy has significantly reduced mother-to-child transmission (MTCT) of HIV, with transmission rates declining to below 1–2% in high-income countries and under 5% in certain low- and middle-income settings. However, persistent challenges, including adherence, retention in care, and equitable access to ART, necessitate continued research and programmatic improvements. Pharmacokinetic changes in pregnancy may impact drug absorption and metabolism, underscoring the need for therapeutic drug monitoring (TDM) and individualized treatment adjustments to ensure optimal drug exposure and virologic suppression. As ART regimens continue to evolve, pregnant women must remain a priority population for treatment optimization, balancing maternal viral suppression with fetal safety [58].

Advancing ART management during pregnancy requires global collaboration to ensure findings are generalizable across diverse populations and healthcare systems. Multinational and multicenter studies facilitate the development of globally applicable guidelines, ensuring equitable access for pregnant individuals living with HIV. Strengthening international partnerships can further support resource-limited regions through capacity-building initiatives, shared research infrastructure, and knowledge exchange programs [58].

Given the multidisciplinary nature of ART optimization, integrating pharmacokinetic insights, TDM, and personalized medicine will be essential in addressing the unique physiological challenges of pregnancy. Future efforts should focus on harmonizing guidelines with real-world applicability, evaluating novel long-acting therapies, and ensuring that ART benefits reach all pregnant individuals, regardless of geographic or socioeconomic barriers. By prioritizing research and fostering collaboration across clinical, pharmacological, and public health disciplines, the field can advance toward more effective, equitable, and sustainable ART strategies, ultimately contributing to the global goal of eliminating MTCT of HIV [58].

Table 2 presents the practical recommendations for dose adjustments and therapeutic monitoring in pregnant women with HIV.

## 7. Conclusions

The pharmacokinetic (PK) adaptations that occur during pregnancy necessitate a tailored approach to antiretroviral therapy (ART) to ensure maternal virological suppression while minimizing the risk of mother-to-child transmission (MTCT). The available evidence highlights significant changes in drug metabolism, distribution, and elimination, which impact the plasma concentrations of various ART agents. While most integrase strand transfer inhibitors (INSTIs) and nucleoside reverse transcriptase inhibitors (NRTIs) maintain efficacy without the need for dose adjustments, certain protease inhibitors (PIs) and cobicistat-boosted regimens require modifications or should be avoided due to subtherapeutic drug exposure. TDM plays a crucial role in mitigating these challenges, particularly for drugs with significant PK variability. Despite these pharmacokinetic alterations, the overall safety of ART in pregnancy is well established, with no substantial increase in adverse neonatal outcomes. However, disparities in access to optimized ART strategies, including TDM and pharmacogenomic testing, continue to pose challenges, particularly in low-resource settings.

Future guidelines should integrate individualized ART management strategies that incorporate TDM, pharmacogenomic profiling, and dose optimization based on pregnancy-specific PK data. Given the growing body of evidence on drug metabolism during pregnancy, harmonization of international ART guidelines is essential to ensure consistent, evidence-based recommendations. Special attention should be given to standardizing protocols for dose adjustments, defining thresholds for therapeutic monitoring, and optimizing ART selection based on population-specific PK profiles.

Priorities for future research should include longitudinal studies evaluating the long-term safety of in utero ART exposure, particularly concerning neurodevelopmental, metabolic, and immunological outcomes. Additionally, comparative studies between newer ART regimens will be instrumental in determining the most effective and safest options for pregnant women. The potential role of long-acting injectable ART in pregnancy warrants further investigation, as it may provide a promising alternative for women with adherence challenges.

From a public policy perspective, expanding access to TDM and pharmacogenomic testing, particularly in low- and middle-income countries, should be prioritized to bridge existing disparities in ART management. Strengthening global collaboration in ART research and implementation, promoting capacity-building initiatives, and supporting cost-effective solutions for ART monitoring will be essential in optimizing HIV care for pregnant women worldwide. Ultimately, ensuring equitable and effective ART strategies will not only improve maternal and neonatal health outcomes but also contribute significantly to global efforts to eliminate pediatric HIV infections.

## Figures and Tables

**Table 1:** CYP substrates, inhibitors, and inducers used in HIV treatment.

Antiretroviral (ARV) Drug Class	CYP Substrate(s)	CYP Inhibitor(s)	CYP Inducer(s)
Protease Inhibitors (PIs)			
Atazanavir (ATV)	CYP3A4	CYP3A4, CYP2C8 (weak)	-
Darunavir (DRV)	CYP3A4	CYP3A4	CYP2C9
Fosamprenavir (FPV)	CYP3A4	CYP3A4	CYP3A4 (weak)
Lopinavir (LPV)	CYP3A4	CYP3A4 (weak)	-
Saquinavir (SQV)	CYP3A4	CYP3A4	-
Tipranavir (TPV)	CYP3A4	CYP3A4, CYP2D6 (weak)	CYP3A4, CYP1A2, CYP2C19
Indinavir (IDV)	CYP3A4	CYP3A4	-
Nelfinavir (NFV)	CYP3A4, CYP2C19	CYP3A4	-
Non-Nucleoside Reverse Transcriptase Inhibitors (NNRTIs)			
Efavirenz (EFV)	CYP2B6 (primary), CYP2A6, CYP3A4	CYP3A4	CYP3A4, CYP2B6, CYP2C19
Etravirine (ETR)	CYP3A4, CYP2C9, CYP2C19	CYP2C9, CYP2C19	CYP3A4
Delavirdine (DLV)	CYP3A4	CYP3A4, CYP2D6, CYP2C9, CYP2C19	-
Nevirapine (NVP)	CYP3A4, CYP2B6	CYP3A4, CYP2B6	-
Rilpivirine (RPV)	CYP3A4	-	-
Nucleoside/Nucleotide Reverse Transcriptase Inhibitors (NRTIs)			
Abacavir (ABC)	Minor CYP involvement	-	-
Tenofovir (TDF, TAF)	Not CYP-metabolized	-	-
Emtricitabine (FTC)	Not CYP-metabolized	-	-
Integrase Strand Transfer Inhibitors (INSTIs)			
Bictegravir (BIC)	CYP3A4	-	-
Dolutegravir (DTG)	CYP3A4 (minor)	-	-
Elvitegravir (EVG)	CYP3A4	CYP2C9	-
Raltegravir (RAL)	Not CYP-metabolized (UGT1A1)	-	-
Cabotegravir (CAB)	Not CYP-metabolized (UGT1A1 and UGT1A9)	-	-
CCR5 Antagonist			
Maraviroc (MVC)	CYP3A4	-	-
Pharmacokinetic Enhancers (Boosters)			
Cobicistat (COBI)	CYP3A4	CYP3A4, CYP2D6	-
Ritonavir (RTV)	CYP3A4, CYP2D6	CYP3A4, CYP2D6	CYP1A2, CYP2B6, CYP2C8, CYP2C9, CYP2C19
Novel ARVs and Long-Acting Agents			
Fostemsavir (FTR)	CYP3A4	-	-
Lenacapavir (LEN)	CYP3A4	-	-
Ibalizumab (IBA)	Not CYP-metabolized	-	-

Table adapted from Gong Y et al. [18].

**Table 2:** Practical recommendations for dose adjustments and therapeutic monitoring in pregnant women with HIV.

Antiretroviral Class	Drug	Dose Adjustment Recommendations	Therapeutic Monitoring Recommendations
Protease Inhibitors (PIs)	Lopinavir/ritonavir (LPV/r)	Increase to 600/150 mg or 500/125 mg twice daily in the second and third trimesters for PI-experienced patients or those with baseline VL > 50 copies/mL. Avoid once-daily dosing.	Monitor plasma concentrations in late pregnancy to ensure therapeutic drug levels.
Protease Inhibitors (PIs)	Atazanavir/ritonavir (ATV/r)	Increase to 400/100 mg in the second and third trimesters if coadministered with tenofovir-DF or an H2-receptor antagonist.	Monitor plasma concentrations, particularly when coadministered with tenofovir.
Integrase Strand Transfer Inhibitors (INSTIs)	Dolutegravir (DTG)	No dose adjustment required in most cases. Close monitoring is advised when coadministered with enzyme inducers like rifampicin.	Monitor trough levels if coadministered with enzyme inducers.
Integrase Strand Transfer Inhibitors (INSTIs)	Elvitegravir/cobicistat (EVG/c)	Avoid use due to significant pharmacokinetic reductions leading to subtherapeutic levels.	Not applicable (drug should be avoided).
Nucleoside Reverse Transcriptase Inhibitors (NRTIs)	Tenofovir disoproxil fumarate (TDF)	Standard dose (300 mg once daily) is generally sufficient. Additional monitoring is recommended in renal impairment.	Monitor renal function and tenofovir levels in renal impairment.
Cobicistat-Boosted Regimens	Elvitegravir/cobicistat (EVG/c), Atazanavir/cobicistat (ATV/c)	Avoid use during pregnancy due to loss of boosting effect, leading to insufficient drug exposure and risk of virological failure.	Not applicable (drug should be avoided).

## Data Availability

Not applicable.

## Supplementary Materials

The following supporting information can be downloaded at https://www.mdpi.com/article/10.3390/pharmaceutics17070913/s1: Table S1: Summary table clinical trials ARV drugs in pregnancy.
