# Supplementary material for: Pharmacokinetic Adaptations in Pregnancy: Implications for Optimizing Antiretroviral Therapy in HIV-Positive Women
Source: Pharmaceutics. 2025 Jul 15;17(7):913. doi: 10.3390/pharmaceutics17070913 (PMC12300238; doi:10.3390/pharmaceutics17070913)
Supplement: Supplementary file 1 [file pharmaceutics-17-00913-s001.zip › pharmaceutics-3518234-supplementary.pdf]

Supplementary Materials: Pharmacokinetic Adaptations in Pregnancy: Implications for Optimizing Antiretroviral Therapy in HIV-Positive Women

Natalia Briceño-Patiño, María Camila Prieto, Paula Manrique, Carlos-Alberto Calderon-Ospina and Leonardo Gómez

Table S1. Summary table clinical trials ARV drugs in pregnancy.

Non-Nucleoside Reverse Transcriptase Inhibitors (NNRTIs)

1.Article	2. Objective	3. Methodological aspects	4. Results	5. Additional observations
Pharmacokinetics, Pharmacodynamics, and Pharmacogenetics of Efavirenz 400 mg Once Daily During Pregnancy and Post-Partum	The study aimed to investigate the pharmacokinetics (PK), efficacy, and pharmacogenetic aspects of using 400 mg of efavirenz once daily in pregnant women with HIV during the third trimester (TT) and the postpartum period (PP). The study sought to determine whether the reduced efavirenz dose (400 mg) could maintain adequate plasma levels for viral suppression and serve as an effective and safe alternative to the standard 600 mg dose in pregnant women, despite the physiological changes of pregnancy.	The study was an open-label, multicenter trial conducted at two sites (United Kingdom and Uganda) and included 25 pregnant women with HIV who had previously been treated with the standard 600 mg dose of efavirenz, tenofovir disoproxil fumarate (TDF), and emtricitabine (FTC) or lamivudine (3TC), but were switched to a 400 mg dose of efavirenz. Therapeutic drug monitoring (TDM) was performed weekly to measure plasma levels of efavirenz, and PK profiles were evaluated during the TT and PP. Genetic variants of the **CYP2B6** gene, which influence efavirenz metabolism, were also analyzed.	Efavirenz levels remained above the therapeutic threshold of 800 ng/mL during both the TT and PP, despite PK parameters being slightly lower during the TT (Cmax decreased by 3%, AUC by 13%, and Ctrough by 23%). All women maintained an undetectable viral load (<50 copies/mL) throughout the study, and none of the infants born to participants acquired HIV. Variants of the **CYP2B6** gene indicated that 5 out of 25 women were slow metabolizers, resulting in higher efavirenz concentrations; however, this did not impact efficacy or cause significant adverse effects. The drug was well-tolerated, with no grade 3 or 4 adverse events, indicating a favorable safety profile for the reduced dose.	A methodological limitation is the relatively small sample size (25 women), which could affect the generalizability of the results. Insufficient cord blood plasma samples were collected at delivery, limiting a comprehensive assessment of the maternal-fetal transfer of the drug. Although **CYP2B6** genetic variants were investigated, the data on the clinical relevance of these variants in the pharmacokinetics of 400 mg efavirenz during pregnancy may not be sufficient for extrapolation to all populations.
Unintended Pregnancies Observed With Combined Use of the Levonorgestrel	Evaluate the effect of coadministration of efavirenz- or nevirapine-based antiretroviral	A non-randomized, parallel-group study conducted in Ugandan	Women who received efavirenz-based antiretroviral therapy had significantly lower	The study was open-label and non-randomized, which may introduce bias. Sexual

Contraceptive Implant and Efavirenz-based Antiretroviral Therapy	therapy on the pharmacokinetics of levonorgestrel in subdermal implants in women with HIV infection.	women infected with HIV. A total of 57 women participated, divided into three groups: ART-naïve group, efavirenz group, and nevirapine group. Levonorgestrel plasma concentrations were measured at different time points over a 48-week period.	levonorgestrel concentrations compared to women who were not receiving antiretroviral treatment (ART-naïve). At 24 weeks, levonorgestrel concentrations were 47% lower, and at 48 weeks, they were 57% lower. In the efavirenz group, there were three unintended pregnancies (15%) over the 48-week period, highlighting a potential contraceptive failure due to the interaction between levonorgestrel and efavirenz. In contrast, women who received nevirapine had 35% higher levonorgestrel concentrations at 24 weeks and 14% higher at 48 weeks, compared to the ART-naïve group. No pregnancies were observed in this group or in the ART-naïve group.	activity of the participants was not assessed, nor were specific measures of contraceptive effectiveness, such as ovulation detection, included.

Protease Inhibitors (PIs)

Lopinavir/ritonavir, atazanavir, darunavir, and others

1.Article	2. Objective	3. Methodological aspects	4. Results	5. Additional observations
Atazanavir exposure is effective during pregnancy regardless of tenofovir use	The aim of the study was to describe the pharmacokinetics of darunavir in HIV-1 infected pregnant women during the third trimester and the postpartum period, and to evaluate its efficacy in preventing mother-to-child transmission of HIV.	**Study Design:** This was a non-randomized, open-label, multicenter (Phase IV) study involving pregnant women infected with HIV, recruited from treatment centers across Europe. **Study Population:** The study included 24 pregnant women who received darunavir/ritonavir (800/100 mg	**Reduction in Darunavir Exposure During Pregnancy:** Compared to the postpartum period, a 22% reduction in AUC was observed for the 600/100 mg twice-daily dose and a 34% reduction for the 800/100 mg once-daily dose. The maximum concentrations (Cmax) were also lower during	**Methodological Limitations:** One limitation of the study was the lack of determination of the free concentration of darunavir across the entire pharmacokinetic curve, as there was insufficient plasma volume for all analyses. Additionally, genotyping data were not collected, preventing an evaluation of

		once daily or 600/100 mg twice daily) as part of their combined antiretroviral therapy (cART).<br>**Measurements:** Pharmacokinetic curves were obtained during the third trimester and postpartum, measuring both total and free darunavir concentrations. Maternal blood and umbilical cord samples were collected to evaluate the transplacental passage of the drug.<br>**Data Analysis:** Pharmacokinetic parameters such as the area under the curve (AUC), maximum concentration (Cmax), and trough concentrations (Ctrough) were calculated for both doses and compared between the third trimester and postpartum period.	pregnancy, with reductions of 24% and 22%, respectively.<br>**Free Fraction of Darunavir:** The free fraction of darunavir, which represents the active portion of the drug, remained constant during pregnancy (12%) and postpartum (10%).<br>**Transplacental Passage:** The transplacental passage of darunavir was low, with a median cord-to-maternal ratio of 0.13, indicating limited fetal transfer.<br>**Prevention of Mother-to-Child HIV Transmission:** No cases of mother-to-child HIV transmission were recorded, and all babies tested negative for HIV. No congenital anomalies were reported.	potential resistance to protease inhibitors.<br>**Irrelevant Findings:** Although the study found that some babies were born with low birth weight for their gestational age, there was insufficient evidence to attribute this finding to the darunavir treatment.
No Need for Lopinavir Dose Adjustment during Pregnancy: a Population Pharmacokinetic and Exposure-Response Analysis in Pregnant and Nonpregnant HIV-Infected Subjects	To evaluate the need for dose adjustments of lopinavir/ritonavir during pregnancy in women living with HIV-1, as a decrease in lopinavir exposure during pregnancy has been reported, but it is unclear whether this has clinical relevance	A population pharmacokinetic analysis was performed using a non-linear mixed effects model to evaluate lopinavir and ritonavir concentrations in plasma.<br>The study included data from six clinical trials comprising a total of 84 pregnant women and 595 nonpregnant HIV-1 subjects. Participants were both treatment-naive and treatment-experienced in antiretroviral therapy.<br>Lopinavir/ritonavir doses ranged from 400/100 mg to 600/150 mg, administered twice	Lopinavir clearance was 17% higher in pregnant women compared to non-pregnant subjects, suggesting increased drug elimination during pregnancy. Postpartum, clearance was 26.4% lower than in non-pregnant subjects.<br>The lopinavir/ritonavir tablet formulation demonstrated 20% higher bioavailability compared to the soft gel capsule formulation. This was significant in maintaining adequate drug levels in pregnant women despite the increased clearance.	Although lopinavir clearance was higher in pregnant women, the greater bioavailability of the tablet formulation (20% higher than the capsule formulation) compensated for this increased clearance. Therefore, no dose adjustment was necessary to maintain therapeutic levels of lopinavir in pregnant women.<br>No clear relationship was observed between lopinavir levels and viral load in pregnant women, suggesting that variations in drug exposure do not significantly affect

		daily.<br>Intensive drug concentration analyses were performed at regular intervals before and after dosing. Multiple pharmacokinetic time points were evaluated, including the second and third trimesters of pregnancy and a postpartum period.	No statistically significant differences were found in lopinavir exposure levels (AUC0-12 and Cpredose) between pregnant women in the second and third trimesters and non-pregnant subjects who received the same drug doses. Similar efficacy was observed between pregnant women and non-pregnant subjects receiving lopinavir/ritonavir at a 400/100 mg dose twice daily, supporting the use of this dose during pregnancy without the need for adjustment.	virological efficacy within the observed range. This supports the notion that the standard dose is effective without the need for adjustments.<br>More than 90% of pregnant women achieved virological suppression (less than 50 copies/mL), indicating that the treatment was effective in this population.
Atazanavir Plus Cobicistat: Week 48 and Week 144 Subgroup Analyses of a Phase 3, Randomized, Double-Blind, Active-Controlled Trial	The objective of the article is to describe the virological response and treatment discontinuation in various subgroups of patients who received atazanavir plus cobicistat (ATV+COBI) compared to atazanavir plus ritonavir (ATV+RTV), both in combination with emtricitabine/tenofovir disoproxil fumarate (FTC/TDF). The aim was to confirm the similarity of the outcomes between both regimens in terms of virological efficacy, virological failures, and discontinuations due to adverse events, while also evaluating these results in patients with diverse demographic and disease characteristics.	**Study Design:** The study is a phase 3, international, randomized, double-blind, double-dummy, active-treatment-controlled clinical trial. It was approved by the ethics committees of all participating centers. Patients were randomly assigned in a 1:1 ratio to two treatment groups: ATV+COBI or ATV+RTV, both in combination with FTC/TDF.<br>**Study Population:** The participants were HIV-1-infected adults, treatment-naive, with HIV-1 RNA levels ≥5000 copies/mL. Patients were selected based on estimated glomerular filtration rate (≥70 mL/min) and genotypic sensitivity to atazanavir,	**Virological Success:**<br>● At 48 weeks, 85.2% of patients treated with atazanavir plus cobicistat (ATV+COBI) achieved virological success (HIV RNA <50 copies/mL), compared to 87.4% of patients treated with atazanavir plus ritonavir (ATV+RTV).<br>● At 144 weeks, 72.1% of patients in the ATV+COBI group and 74.1% in the ATV+RTV group achieved virological success.<br>● No significant differences were found between the two regimens overall, although women receiving ATV+COBI at 144 weeks had a higher virological success rate (OR 2.36; 95% CI: 1.02, 5.47).<br>**Virological Failure:**	**Methodological Limitations:**<br>● **Unpowered Post Hoc Analysis:** The subgroup analyses were post hoc and were not initially designed to assess differences between subgroups. This means that the results obtained in the subgroups do not have the same level of robustness as the primary analyses. Furthermore, no adjustments were made for multiple comparisons, which increases the risk of finding statistically significant results by chance.<br>● **Imbalance in Events Such as Pregnancies:** The higher rate of pregnancies in the ATV+RTV group may have influenced the results in women, introducing bias in the findings related to this subgroup. This

	emtricitabine, and tenofovir at screening.<br>**Subgroup Evaluation:** Post hoc analyses assessed virological response and discontinuation due to adverse events in subgroups defined by:<br>• CD4 count (≤200, 201-350, >350 cells/mm³),<br>• HIV-1 RNA levels in blood (≤100,000 and >100,000 copies/mL),<br>• Race (White, Black/African American, Asian, and other),<br>• Age (<40 years and ≥40 years),<br>• Sex (male and female).<br>**Statistical Analysis:** Univariate odds ratios (OR) and their 95% confidence intervals were used to compare the proportion of virological success (HIV RNA <50 copies/mL) and discontinuation due to adverse events across subgroups. The proportions of patients with virological success and failure were analyzed using the FDA "snapshot" algorithm.<br>**Follow-up Duration:** Results were evaluated at two time points, at weeks 48 and 144 of treatment.	• At 48 weeks, 5.8% of patients in the ATV+COBI group experienced virological failure, compared to 4.0% in the ATV+RTV group.<br>• At 144 weeks, the virological failure rates were 8.1% and 4.9% for the ATV+COBI and ATV+RTV groups, respectively.<br>**Discontinuation Due to Adverse Events (AEs):**<br>• Discontinuation rates due to AEs were not significantly different between the two groups at 48 and 144 weeks.<br>○ At 48 weeks, 7.3% of patients in the ATV+COBI group and 7.2% in the ATV+RTV group discontinued treatment due to AEs.<br>○ At 144 weeks, the rates were 11.0% and 11.2%, respectively.<br>**Specific Subgroups:**<br>• No significant differences in virological success or discontinuation due to AEs were observed in most subgroups, including different CD4 count ranges, HIV-1 RNA levels, race, sex, and age.<br>• An exception was virological success in women at 144 weeks, where ATV+COBI showed better results than ATV+RTV, driven by fewer discontinuations due to pregnancy and withdrawals of consent in the ATV+COBI group.	imbalance appears to be attributable to chance, but the reasons behind this phenomenon were not thoroughly explored.<br>**Irrelevant Findings:**<br>• **Interruptions Due to Pregnancies:** The higher rate of interruptions due to pregnancies in the ATV+RTV group influenced the results in the female subgroup, but this does not reflect a real difference in the efficacy of the treatments. Rather, it highlights the need to closely monitor contraception in trials involving women of reproductive age, rather than suggesting a clinical advantage of ATV+COBI over ATV+RTV.

Pharmacokinetics of once-daily darunavir/ritonavir in HIV-1–infected pregnant women	To evaluate the influence of pregnancy (during the second and third trimesters of gestation) on the pharmacokinetics of once-daily (QD) darunavir/ritonavir, compared to the postpartum period. Secondary objectives included documenting the antiviral activity, safety, and tolerability of the QD darunavir/ritonavir regimen during pregnancy and postpartum, comparing darunavir/ritonavir concentrations between maternal plasma and umbilical cord plasma at delivery, and assessing neonatal outcomes in infants born to women treated with this regimen during pregnancy.	**Study Design:** This was a phase IIIb, multicenter, open-label, single-arm trial conducted at 11 sites in the United States and one site in Puerto Rico.<br>**Participants:** Pregnant women living with HIV-1, aged 18 years or older, between 18 and 26 weeks of gestation, were included. Participants were receiving an antiretroviral regimen containing once-daily darunavir/ritonavir 800/100 mg.<br>**Pharmacokinetic Evaluations:** Pharmacokinetic analyses were conducted over 24 hours during three visits:<br>● **Second trimester** (24–28 weeks of gestation).<br>● **Third trimester** (34–38 weeks of gestation).<br>● **Postpartum** (6–12 weeks postpartum).<br>Plasma concentrations of darunavir and ritonavir were measured using high-performance liquid chromatography coupled with tandem mass spectrometry (HPLC-MS/MS).<br>**Parameters Evaluated:**<br>● Total and unbound (active fraction) concentrations of darunavir and ritonavir.<br>● Pharmacokinetic parameters, including area under the curve (AUC24h), minimum concentration (Cmin), maximum concentration (Cmax), and time	**Decreased Darunavir Exposure During Pregnancy:**<br>● Total darunavir exposure (measured as AUC24h) was 34–35% lower during pregnancy (second and third trimesters) compared to the postpartum period.<br>● The maximum concentration (Cmax) of darunavir was 31–34% lower during pregnancy.<br>● The minimum concentration (Cmin) of darunavir was 32–50% lower during pregnancy.<br>**Unbound Darunavir Exposure (Active Fraction):**<br>● Unbound darunavir exposure was reduced, though the decrease was less pronounced than that of total darunavir, with a 20–24% reduction in AUC24h during pregnancy.<br>● The Cmin of unbound darunavir was 13–38% lower during pregnancy.<br>**Antiviral Efficacy:**<br>● The percentage of women with an undetectable viral load (<50 copies of HIV-1 RNA/mL) increased from 59% at baseline to 87–100% during pregnancy and postpartum.<br>● All infants born to the 16 women who completed the study tested HIV-negative.<br>The treatment was well tolerated, with only one serious adverse	The study included 17 pregnant women.<br>While the data obtained are valuable, the small sample size may limit the generalizability of the results.<br>Although medication adherence was assessed through self-reporting, no objective method was used. This leaves the possibility that fluctuations in plasma darunavir levels may be linked to undetected suboptimal adherence.<br>While some adverse events were reported, the follow-up period was relatively short, potentially limiting the identification of long-term effects on both mothers and infants.<br>As a single-arm study, no comparison group was included (e.g., a group receiving an alternative treatment regimen or a different dosing schedule). A control group could have provided better context for interpreting the results.<br>Although consistent with previous studies, the low placental transfer of darunavir and ritonavir may be relevant in scenarios where greater fetal protection is desired and should be considered in therapeutic decision-making.<br>Dose adjustments for lopinavir (LPV) were not recommended, as free LPV concentrations were adequate in

		to maximum concentration (tmax).	event (gestational diabetes) possibly related to the study medication.<br>Darunavir concentrations in umbilical cord plasma were low compared to maternal plasma concentrations, with median values of 12.05% for darunavir and 7.70% for ritonavir, indicating low placental transfer.	both pregnant and non-pregnant women.
Reduced exposure to darunavir and cobicistat in HIV-1-infected pregnant women receiving a darunavir/cobicistat-based regimen	To evaluate the influence of pregnancy (during the second and third trimesters) on the pharmacokinetics of once-daily (QD) darunavir/ritonavir compared to the postpartum period. Secondary objectives included documenting the antiviral activity, safety, and tolerability of the darunavir/ritonavir QD regimen during pregnancy and postpartum, comparing darunavir/ritonavir concentrations between maternal plasma and umbilical cord plasma at delivery, and assessing outcomes in infants born to women treated with this regimen during pregnancy.	**Study Design:** The study was a phase IIIb, multicenter, single-arm, open-label trial conducted at 11 sites in the United States and one site in Puerto Rico.<br>**Participants:** Pregnant women living with HIV-1, aged over 18 years, with 18 to 26 weeks of gestation, participated. Women receiving an antiretroviral regimen that included once-daily darunavir/ritonavir 800/100 mg were eligible.<br>**Pharmacokinetic Evaluations:** Pharmacokinetic analyses were conducted over a 24-hour period during three visits:<br>● **Second trimester:** 24-28 weeks of gestation.<br>● **Third trimester:** 34-38 weeks of gestation.<br>● **Postpartum:** Between 6 and 12 weeks after delivery.<br>Darunavir and ritonavir plasma concentrations were measured using high-performance liquid	**Reduction in Darunavir Exposure During Pregnancy:**<br>● Total darunavir exposure (measured as AUC24h) was 34-35% lower during pregnancy (second and third trimesters) compared to the postpartum period.<br>● Maximum concentration (Cmax) of darunavir was 31-34% lower during pregnancy.<br>● Minimum concentration (Cmin) of darunavir was 32-50% lower during pregnancy.<br>**Unbound Darunavir Exposure (Active Fraction):**<br>● Unbound darunavir exposure was reduced, but the decrease was less pronounced than that of total darunavir, with a 20-24% reduction in AUC24h during pregnancy.<br>● Unbound darunavir Cmin was 13-38% lower during pregnancy.<br>**Antiviral Efficacy:**	**Limited Sample Size:** The study included 17 pregnant women, and while the data obtained are valuable, the small sample size may limit the generalizability of the results.<br>**Lack of Treatment Compliance Monitoring:** Although adherence to medication was assessed through self-reporting, no objective methods were used, leaving the possibility that fluctuations in darunavir plasma levels could be related to undetected suboptimal adherence.<br>**Limitations in Adverse Event Assessment:** While some adverse events were reported, the relatively short follow-up period may limit the identification of long-term effects on both mothers and infants.<br>**Absence of a Control Group:** As a single-arm study, there was no comparison group (e.g., a group receiving a different treatment regimen or an alternative dosing schedule),

		chromatography coupled with tandem mass spectrometry (HPLC-MS/MS).<br>**Evaluated Parameters:**<br>● Total and unbound (active fraction) concentrations of darunavir and ritonavir.<br>● Pharmacokinetic parameters, including area under the curve (AUC24h), minimum concentration (Cmin), maximum concentration (Cmax), and time to reach maximum concentration (tmax).	● The percentage of women with an undetectable viral load (< 50 copies of HIV-1 RNA/mL) increased from 59% at baseline to 87-100% during pregnancy and postpartum.<br>● All infants born to the 16 women who completed the study were HIV-negative.<br>**Safety:**<br>● The treatment was well tolerated, with only one serious adverse event (gestational diabetes) possibly related to the study medication.<br>● No serious adverse events related to the treatment were reported in the infants.<br>**Placental Transfer:**<br>● Darunavir concentrations in umbilical cord plasma were low compared to maternal plasma concentrations, with a median of 12.05% for darunavir and 7.70% for ritonavir, indicating low placental transfer.	which could have provided better context for the results.<br>**Low Placental Transfer:** While consistent with previous studies, the low placental transfer of darunavir and ritonavir may be relevant in scenarios where greater fetal protection is desired and should be considered in therapeutic decision-making.
Population Approach To Analyze the Pharmacokinetics of Free and Total Lopinavir in HIV-Infected Pregnant Women and Consequences for Dose Adjustment	To describe the pharmacokinetics of total and free lopinavir (LPV) in pregnant women living with HIV to assess the need for dose adjustments during pregnancy. The study also evaluated the placental transfer of LPV and the impact of different genetic polymorphisms.	Maternal plasma, umbilical cord blood, and amniotic fluid samples from 208 women were analyzed.<br>Two population pharmacokinetic models were developed using NONMEM software: one to describe the pharmacokinetics of total and free lopinavir (LPV) and another to evaluate the placental transfer of LPV.<br>The study included 208 pregnant women living with HIV, some	A 39% increase in total lopinavir (LPV) clearance was observed in pregnant women compared to non-pregnant women. Despite this increased clearance, free LPV concentrations (the unbound fraction) in pregnant women were comparable to those in non-pregnant women.<br>Free LPV concentrations, which are critical for antiviral activity, did not decrease significantly during	No dose adjustments for LPV were recommended during pregnancy, as free LPV concentrations were adequate in both pregnant and non-pregnant women.<br>**Limitations:**<br>Sampling times varied among participants, which may have affected some measurements. Fetal polymorphisms were not studied, which could have been useful for better

		receiving monotherapy with lopinavir/ritonavir (LPV/r) and others on triple therapy with LPV/r plus two nucleoside reverse transcriptase inhibitors (NRTIs). Non-pregnant women were also included for comparison. A total of 400 maternal plasma samples, 79 umbilical cord blood samples, and 48 amniotic fluid samples were collected. LPV concentrations were measured in these samples in both total and free (unbound to plasma proteins) forms (26149996). Several genetic polymorphisms were analyzed for their potential influence on LPV pharmacokinetics, including genes involved in drug metabolism (CYP3A5, CYP3A4) and placental transporters (P-gp, SLCO1B1).	pregnancy, even with the observed increase in clearance. This suggests that free LPV concentrations remained adequate during pregnancy, indicating no need to increase LPV dosing in pregnant women. Placental transfer of LPV was low, with an estimated fetal-to-maternal ratio of 11.6%, confirming that only a small amount of LPV crosses the placenta and reaches the fetus. The evaluated genetic polymorphisms (CYP3A5, CYP3A4, P-gp, SLCO1B1) did not have a significant impact on LPV pharmacokinetics or placental transfer of the drug. No significant differences in LPV pharmacokinetics were found between women receiving monotherapy (LPV/r alone) and those on triple therapy (LPV/r plus two NRTIs).	understanding placental transfer.
Impact of body weight and missed doses on lopinavir concentrations with standard and increased lopinavir/ritonavir doses during late pregnancy	To evaluate the influence of body weight and missed doses on the pharmacokinetics of lopinavir using standard and increased doses of lopinavir/ritonavir in pregnant women during the third trimester.	The study analyzed plasma concentration data of lopinavir obtained from clinical trials in HIV-infected pregnant women in Thailand and the United States, using non-linear mixed-effects regression models and Monte Carlo simulations to estimate the probability of achieving therapeutic concentrations of lopinavir.	The standard dose of 400/100 mg of lopinavir/ritonavir is sufficient for most pregnant women to maintain therapeutic concentrations of lopinavir during the third trimester. The risk of subtherapeutic concentrations is higher in women with a body weight greater than 100 kg. Approximately 7% of women weighing over 100 kg may not reach therapeutic concentrations	The study highlights that the standard dose may be insufficient in women with high body weight and also emphasizes the impact of missed doses, suggesting that an increased dose may be more effective in preventing subtherapeutic concentrations in these circumstances.

			using the standard dose. With an increased dose of 600/150 mg, this risk is significantly reduced to less than 2%, indicating that this dose is more appropriate for women with higher body weight or a history of poor treatment adherence.	

Integrase Strand Transfer Inhibitors (INSTIs)

Detailed reviews for dolutegravir, raltegravir, bictegravir, etc.

1.Article	2. Objective	3. Methodological aspects	4. Results	5. Additional observations
CROI 2023: Advances in Antiviral Therapy in HIV and Viral Hepatitis	The main objective of the article is to review the advances in antiviral therapy for HIV and viral hepatitis presented at the 2023 Conference on Retroviruses and Opportunistic Infections (CROI). The focus is on improving diagnosis, linkage to care, and viral suppression, particularly in vulnerable populations, as well as evaluating new treatments and strategies for coinfection with hepatitis B (HBV) and hepatitis C (HCV).	It includes both clinical trials and observational studies related to antiviral therapy for HIV and hepatitis. Some studies employ randomized controlled trial (RCT) designs, such as the use of point-of-care (POC) testing to improve diagnosis in infants, while others utilize pragmatic models and cohort studies. The included studies vary in their approaches but focus on long-acting antiretroviral treatment, the management of HIV coinfections with HBV and HCV, as well as specific populations such as pregnant women and adolescents.	Long-acting therapies: Promising data were reported on the use of long-acting cabotegravir and rilpivirine, demonstrating similar efficacy to oral regimens in viral suppression of HIV. Hepatitis B coinfection: Studies indicated that the TAF/FTC/BIC regimen is superior to TDF/FTC/DTG in suppressing HBV in individuals coinfected with HIV. Pharmacokinetics during pregnancy: Plasma concentrations of bictegravir were reduced during pregnancy, potentially compromising the efficacy of this regimen in pregnant women. Additionally, interactions between antiretroviral therapy and hormonal contraception were reported. Impact of the COVID-19 pandemic: A decrease in HIV viral suppression and	Serious methodological shortcomings: Some limitations include smaller than expected sample sizes in certain studies, such as the evaluation of point-of-care (POC) testing for infants in Mozambique and Tanzania, which complicated the assessment of overall impact. Other studies faced challenges in long-term evaluation due to interruptions caused by the COVID-19 pandemic, which may have affected the validity of some results. Irrelevant findings: While the review includes a broad range of studies, some data presented, such as those related to therapies still in early phases, may not be applicable in the short term for clinical implementation. The relevance of certain findings related to pharmacokinetics of specific drugs in non-

			patient retention in care was observed during the pandemic, disproportionately affecting vulnerable groups such as people who inject drugs and ethnic minorities. Interventions in vulnerable populations: Several interventions targeting adolescents, pregnant women, and people who inject drugs showed improvements in linkage to care and viral suppression, but face unique challenges such as partner violence and treatment adherence barriers	pregnant populations may not have immediate implications for specific groups, such as pregnant women or individuals with severe coinfections.
Raltegravir in HIV-1-Infected Pregnant Women: Pharmacokinetics, Safety, and Efficacy	The main objective of the study was to evaluate the pharmacokinetics, safety, and efficacy of raltegravir in pregnant women infected with HIV-1. Specifically, the study investigated the impact of physiological changes during pregnancy on drug exposure and its role in preventing mother-to-child transmission (MTCT) of HIV.	The study is a phase IV, multicenter, open-label trial involving 22 pregnant women infected with HIV, all receiving 400 mg of raltegravir twice daily as part of their combined antiretroviral therapy (cART). Pharmacokinetic data were collected at two time points: during the third trimester of pregnancy (around week 33 of gestation) and during the postpartum period (4–6 weeks after delivery). Plasma concentrations of raltegravir were measured at regular intervals over a 12-hour period following drug administration. Additionally, raltegravir levels in umbilical cord blood were assessed to measure placental transfer. Efficacy was assessed by measuring maternal viral	Pharmacokinetics: During the third trimester, raltegravir exposure, measured as area under the curve (AUC), was 29% lower compared to the postpartum period. The maximum concentration (Cmax) decreased by 18%, and the trough concentration (C12h) decreased by 36%. Despite this reduction, the decrease was considered not clinically significant. Placental transfer: The study demonstrated effective placental transfer of raltegravir, with a median cord blood-to-maternal blood ratio of 1.21. Virological efficacy: 86% of the women achieved an undetectable viral load (<50 copies/mL) prior to delivery, and no cases of mother-to-child	Pharmacokinetic variability: One of the key findings was the high interindividual variability in raltegravir exposure. Although the AUC and C12h were reduced on average, some women experienced increases in drug exposure. This variability could be attributed to interactions with other medications or individual differences in maternal physiology. Methodological limitations: Small sample size: With only 22 participants, the study has a small sample size, limiting the ability to generalize the findings. Lack of long-term analysis: The long-term effects of raltegravir exposure on newborns were not studied, and neonatal blood samples

		loads at the end of pregnancy and evaluating the HIV infection status of the newborns.	transmission of HIV were reported. Safety: Raltegravir was well tolerated during pregnancy. No significant congenital abnormalities were reported, and the adverse events observed were not considered severe or related to the use of raltegravir.	were not included to assess postnatal pharmacokinetics. No findings were observed that were irrelevant to the study objectives; however, some minor adverse events were not considered clinically significant or related to the treatment.
Elvitegravir/cobicistat pharmacokinetics in pregnant and postpartum women with HIV	The main objective of the study was to evaluate the pharmacokinetics of darunavir and cobicistat during pregnancy in comparison to the postpartum period, as well as to analyze newborn exposure to these drugs through post-delivery wash samples. The aim was to determine whether standard doses of these medications provided adequate exposure to prevent virological failure and perinatal HIV transmission.	The study was a phase IV, open-label, non-randomized, multicenter clinical trial conducted at several institutions in the United States. A total of 29 pregnant women with HIV participated, all of whom were administered a fixed combination of 800 mg of darunavir and 150 mg of cobicistat once daily. Intensive 24-hour pharmacokinetic profiles were collected during the second and third trimesters of pregnancy and the postpartum period. Liquid chromatography with tandem mass spectrometry (LC-MS/MS) was used to measure the plasma concentrations of darunavir and cobicistat.	Exposure levels to darunavir and cobicistat were significantly lower during pregnancy compared to the postpartum period. Darunavir: The AUC was 53% lower in the second trimester and 56% lower in the third trimester. Cobicistat: The AUC was 50% lower in the second trimester and 56% lower in the third trimester. Placental transfer of both drugs was minimal. Most newborns had undetectable plasma concentrations of the medications, indicating low fetal exposure. Although most women maintained viral suppression, some experienced adverse events, including preterm labor, which may have been related to the treatment.	Methodological shortcomings: The lack of a control group or randomization limits more rigorous comparisons. The selection of women already receiving treatment may introduce bias, as only those responding favorably to the treatment were included. This could have led to an overestimation of positive outcomes and an underestimation of adverse events. The neonatal wash samples were limited, and the sampling time points were broad, which may have affected the accurate assessment of drug elimination in newborns. The results may be difficult to generalize due to these methodological limitations.
Dolutegravir pharmacokinetics in pregnant and postpartum women living with HIV	The main objective of the study is to evaluate the pharmacokinetics of dolutegravir in pregnant women and during the postpartum period, comparing drug concentrations during the second and third	The study is a non-randomized, open-label, multicenter phase IV clinical trial. The participants were 29 pregnant women with HIV who were taking 50 mg of dolutegravir once daily as	Dolutegravir exposure (AUC, Cmax, and C24) was significantly lower during pregnancy compared to the postpartum period. The AUC0-24 was 29% lower in the third trimester and 37% lower	Serious methodological shortcomings: The study relied on opportunistic recruitment of women who were already taking dolutegravir, which could bias the results toward the inclusion of women

	trimesters of pregnancy with those in the postpartum period, and measuring newborn exposure through placental transfer. The study also aimed to determine whether dolutegravir concentrations during pregnancy are sufficient to maintain viral suppression and prevent perinatal HIV transmission.	part of their routine antiretroviral therapy. Intensive pharmacokinetic profiles were collected during the second and third trimesters of pregnancy and between 6-12 weeks postpartum. Dolutegravir concentrations were measured using liquid chromatography-tandem mass spectrometry (LC-MS/MS). In the newborns, umbilical cord blood samples were collected at birth, along with several post-birth samples to assess drug elimination.	in the second trimester compared to postpartum. Despite this reduction, dolutegravir concentrations during pregnancy remained above the EC90 (effective concentration at 90%), ensuring HIV suppression in most women. The cord blood-to-maternal plasma concentration ratio was 1.25, indicating that dolutegravir crosses the placenta efficiently. Dolutegravir elimination in newborns was prolonged, with a half-life of 32.8 hours, more than double that observed in non-pregnant adults. None of the 29 newborns were infected with HIV, and no severe adverse effects related to dolutegravir were reported in either the mothers or babies. However, renal abnormalities were observed in two babies, possibly related to drug exposure.	who were already responding well to treatment and had no adverse effects. This could lead to an underestimation of the risk of toxicities or treatment failures. Irrelevant findings: Some clinical findings in the newborns, such as certain congenital abnormalities, were not considered directly related to dolutegravir exposure.
Twice-Daily Dosing of Dolutegravir in Infants on Rifampicin Treatment: A Pharmacokinetic Substudy of the EMPIRICAL Trial	The objective of the article is to evaluate the pharmacokinetics of dolutegravir in infants with HIV who are receiving dolutegravir twice daily (BID) in combination with rifampin (used to treat tuberculosis) and to compare the results with those who are receiving dolutegravir once daily (OD) without rifampin. The study aims to determine whether the BID	**Study Design**: This was a non-randomized, multicenter pharmacokinetic sub-study with descriptive analysis of safety and efficacy. The sub-study is part of the EMPIRICAL clinical trial, which evaluates empirical treatment for cytomegalovirus and tuberculosis in infants with HIV. **Participants**: Infants aged 1 to 12 months, weighing at least 3 kg, were included.	**Pharmacokinetics of Dolutegravir**: The twice-daily dosing (BID) of dolutegravir in combination with rifampin provided adequate drug exposure. The geometric mean ratios (GMRs) between the groups receiving BID dolutegravir with rifampin and those receiving once-daily (OD) dolutegravir without rifampin were: • Area under the curve (AUC): 0.91 (95%	**Small Sample Size in the Control Group**: The study had a small sample size in the control group (infants not receiving rifampin), with only 6 participants, which may limit the generalizability of the results and make direct comparisons between the two groups difficult. **Self-Reported Treatment Adherence**: Adherence to treatment was self-reported

	dosing with rifampin provides adequate exposure to dolutegravir in these patients and assess the safety and efficacy of the combined treatment in infants with HIV and tuberculosis co-infection.	Participants received dolutegravir twice daily (BID) with rifampin or once daily (OD) without rifampin. Infants were recruited from hospitals in Mozambique, Uganda, Zambia, and Zimbabwe.<br>**Inclusion/Exclusion Criteria**: Infants must have received dolutegravir for at least 14 days and rifampin for at least 30 days. Exclusion criteria included the use of drugs interacting with dolutegravir, severe anemia, or vomiting within 4 hours of medication administration.<br>**Samples and Pharmacokinetic Analysis**: Six blood samples were taken within a 12-hour period for the BID dosing or a 24-hour period for the OD dosing to analyze pharmacokinetic parameters of dolutegravir, such as area under the curve (AUC), maximum concentration (Cmax), and minimum concentration (Ctrough).<br>**Evaluated Parameters**: Plasma levels of dolutegravir, adverse events (AEs), and HIV viral load in infants were evaluated. The safety and efficacy of the treatment were analyzed.<br>**Statistical Analysis**: Pharmacokinetic parameters were determined using non-compartmental analysis, and geometric mean	confidence interval: 0.59-1.42)<br>• Minimum concentration (Ctrough): 0.95 (95% CI: 0.57-1.59)<br>• Maximum concentration (Cmax): 0.87 (95% CI: 0.57-1.33)<br>Only one infant (5%) in the rifampin group had a dolutegravir concentration below the minimum effective level (0.32 mg/L), but none were below the critical threshold (0.064 mg/L).<br>**Safety**:<br>A total of 82 adverse events (AEs) were reported, with 5 possibly related to the treatment (rifampin or dolutegravir). These included liver function abnormalities, all of which resolved without treatment interruption.<br>Two infants (10%) experienced severe adverse events (SAEs) possibly related to rifampin, which also resolved without discontinuing treatment.<br>**Virological Efficacy**:<br>After completing tuberculosis treatment, 76% of infants in the rifampin group and 100% in the non-rifampin group had a viral load of HIV below 1000 copies/mL.<br>The viral load was undetectable in 35% of infants with rifampin and 20% without rifampin by the end of the study.<br>**Conclusion**:<br>Twice-daily dosing of dolutegravir in combination with rifampin	by caregivers, which introduces a potential source of bias. Suboptimal adherence could have affected the pharmacokinetic and efficacy results, particularly with respect to viral load levels.<br>**Limited Virology Data**:<br>Although the pharmacokinetic data for dolutegravir was adequate, the percentage of infants with undetectable viral load was relatively low (35% in the rifampin group and 20% in the non-rifampin group). However, the study attributed this to factors such as the time required for viral suppression in infants, which may not be entirely related to the drug's pharmacokinetics.<br>**Real-World Conditions**:<br>A positive aspect is that the study was conducted under real-world conditions, with infants receiving dolutegravir as part of standard treatment rather than an investigational product. However, this also means that not all factors, such as infant feeding, which could influence drug absorption levels, were strictly controlled.<br>**Limitations in Rifampin Variability**:<br>Higher doses of rifampin are currently being considered for use in children, which could

		ratios were calculated to compare the groups. Descriptive analysis was used for adverse events and virological outcomes.	was well-tolerated and resulted in adequate drug exposure, supporting the use of this dosing strategy for infants with HIV and tuberculosis coinfection.	affect the results. If higher doses of rifampin are implemented, the interaction between dolutegravir and rifampin might be more pronounced than what was observed in this study. **Inequality in Tuberculosis Diagnosis**: In the rifampin group, some infants received treatment without a confirmed diagnosis of tuberculosis, which may have influenced the group comparison. This complicates the comparative analysis between those receiving rifampin and those not receiving it.
Infant Exposure to Dolutegravir Through Placental and Breast Milk Transfer: A Population Pharmacokinetic Analysis of DolPHIN-1	The objective of the article is to develop a population pharmacokinetic model to describe the disposition of dolutegravir in maternal plasma (before and after delivery), umbilical cord plasma, breast milk, and the plasma of breastfed infants 1–3 days after cessation of dolutegravir treatment in mothers living with HIV from the DolPHIN-1 study. Additionally, the potential effects of covariates in mothers and their infants were evaluated to better understand the pharmacokinetics of dolutegravir in this context.	**Study Design:** The study is part of the DolPHIN-1 clinical trial, an open-label, randomized study involving pregnant women living with HIV diagnosed in late pregnancy (28–36 weeks of gestation). Participants were recruited in Uganda and South Africa. **Treatment Allocation:** Pregnant women were randomized 1:1 to receive either dolutegravir-based therapy (50 mg/day) or efavirenz-based therapy. Intensive pharmacokinetic sampling of dolutegravir was conducted in maternal plasma during the third trimester and postpartum, as well as in umbilical cord and breast milk samples.	**Pharmacokinetic Model:** Dolutegravir in maternal plasma was described using a two-compartment model linked to a fetal compartment (umbilical cord) and a breast milk compartment. For infants, dolutegravir was described using a one-compartment model with transfer via breast milk and subsequent elimination. **Placental Transfer:** High placental transfer of dolutegravir was observed, with an umbilical cord-to-maternal plasma ratio of 1.279, indicating efficient transfer of the drug to the fetus. **Breast Milk Exposure:** Dolutegravir concentrations in breast milk were relatively low, with a breast milk-to-maternal plasma ratio	**Sample Size:** Although the study included 28 women and 22 infants, the sample size is relatively small for drawing definitive conclusions, especially considering the potential variability in larger and more diverse populations. **Limitations in Umbilical Cord and Breast Milk Sampling:** There were limitations in the number of umbilical cord and breast milk samples, which reduced the model's ability to accurately estimate inter-individual variability. Only one umbilical cord sample was collected per participant, preventing a detailed assessment of variability. **Assumptions About Dosing and Delivery Times:** Due to the lack

Pharmacokinetic Modeling: A nonlinear mixed-effects model (NONMEM) was used to describe the pharmacokinetics of dolutegravir in maternal plasma, umbilical cord plasma, breast milk, and infant plasma. This model allowed for the evaluation of inter-individual variability in pharmacokinetics and estimation of dolutegravir elimination and distribution parameters in mothers and infants.
Sampling: Maternal plasma samples were collected 14 days after treatment initiation, within 2 weeks postpartum, and in paired umbilical cord samples at delivery. Breast milk and infant plasma samples were also collected after the cessation of maternal dolutegravir treatment.
Covariate Analysis: Covariates were assessed to explain variability in maternal and infant pharmacokinetics. However, no significant effects of covariates were identified on key model parameters.
Pharmacokinetic Predictions: Simulations were conducted to estimate dolutegravir exposure in different matrices (maternal plasma, umbilical cord plasma, breast milk, and infant plasma) and to evaluate the prophylactic protection of of 0.033. This suggests minimal exposure of infants to dolutegravir through breastfeeding.
Infant Exposure: While the contribution of breast milk to infant dolutegravir exposure was low, transplacental exposure provided an average of 4.5 additional days of prophylaxis for some infants after maternal treatment cessation.
Dolutegravir Elimination in Infants: The estimated half-life of dolutegravir in infants was 37.9 hours, indicating slower elimination compared to adults. This is likely due to the immaturity of the infant's enzymatic system, which affects drug metabolism.
Prophylactic Coverage: After maternal dolutegravir treatment was discontinued, some infants maintained drug levels sufficient to provide additional prophylaxis (up to 5.4 days in some cases). However, this protection diminished over time as transplacental dolutegravir was eliminated from the infant's system.
Variability in Pharmacokinetic Parameters: No significant effects of covariates were found on key pharmacokinetic parameters. This suggests that the demographic and clinical factors evaluated did not significantly of precise information in some cases, assumptions were made regarding dosing times and the exact timing of delivery. These assumptions may have introduced biases in the pharmacokinetic model estimates.
Limited Covariate Evaluation: Although some covariates were evaluated, the study found no significant associations with pharmacokinetic parameters. This may be partially due to the small sample size and insufficient data for certain compartments, such as the umbilical cord and breast milk.
Short Follow-Up Duration: The study's follow-up period was limited to the first 1–3 weeks postpartum, preventing a longer-term evaluation of dolutegravir's pharmacokinetic behavior in mothers and infants, as well as potential transmission through breastfeeding over time.
Generalizability of Results: Since participants were recruited from Uganda and South Africa, the findings may not be directly generalizable to populations in other regions of the world with different socioeconomic contexts and health characteristics.
Limited Impact of Breast Milk: While it was demonstrated that

		dolutegravir in infants following maternal treatment cessation.	influence dolutegravir disposition.	dolutegravir exposure through breast milk was low, the clinical impact of this exposure was not explored in depth, particularly in terms of HIV transmission prevention or the development of resistance in exposed infants.
Effect of dihydroartemisinin/piperaquine for malaria intermittent preventive treatment on dolutegravir exposure in pregnant women living with HIV	The aim of the article is to investigate the effect of the dihydroartemisinin/piperaquine combination on the plasma exposure of dolutegravir in pregnant women receiving dolutegravir-based antiretroviral therapy (ART). This is important due to the need for malaria prevention treatments in pregnant women living with HIV in areas of high malaria transmission.	**Study Design:** This was an open-label, non-randomized, fixed-sequence pharmacokinetic study conducted in pregnant women living with HIV in Malawi. **Study Population:** The study included 12 pregnant women in their second or third trimester who were receiving dolutegravir-based antiretroviral therapy (ART). **Intervention:** Participants were given a 3-day treatment dose of dihydroartemisinin/piperaquine, and plasma concentrations of dolutegravir were measured before and after administration. **Pharmacokinetic Analysis:** A non-compartmental analysis was performed to compare dolutegravir pharmacokinetic parameters between the two treatment periods. Geometric mean ratios (GMR) and 90% confidence intervals were calculated to evaluate changes in dolutegravir exposure. **Blood Samples:** Blood samples were collected at multiple time points	**Increase in Dolutegravir Exposure:** Co-administration of dihydroartemisinin/piperaquine increased overall dolutegravir exposure by 30% (GMR 1.30; 90% CI: 1.11–1.52) and the maximum concentration (Cmax) by 31% (GMR 1.31; 90% CI: 1.13–1.51). The trough concentration (C24) of dolutegravir increased by 42% (GMR 1.42; 90% CI: 1.09–1.85). **Tolerability:** The combination of dihydroartemisinin/piperaquine with dolutegravir was well-tolerated. No serious adverse events were observed, with only mild to moderate effects such as nausea and pruritic rashes, which resolved without complications. **Efficacy of Dolutegravir Treatment:** All participants maintained HIV viral loads below 50 copies/mL throughout the study, indicating that dolutegravir treatment remained effective despite the increase in exposure. **Impact of Concomitant Isoniazid Use:** Among participants	**Small Sample Size:** The study included only 12 participants who completed all phases of the analysis, limiting the generalizability of the results. A small sample size reduces the ability to detect smaller differences or accurately assess long-term safety. **Implications of Isoniazid Use:** Although the study explored the impact of isoniazid on dolutegravir exposure, it was not designed to comprehensively evaluate interactions between dolutegravir, dihydroartemisinin/piperaquine, and isoniazid. The small number of participants on isoniazid therapy hindered a robust assessment of this aspect. **Lack of Long-Term Assessment:** The study did not investigate potential long-term effects of the mild increase in dolutegravir exposure, such as possible impacts on maternal weight gain or the development of metabolic syndrome. This is a limitation, as modest changes in drug

		to measure plasma dolutegravir concentrations before and after co-administration with dihydroartemisinin/piperaquine. **Safety Monitoring:** Participants were monitored for 28 days after dihydroartemisinin/piperaquine administration to assess any adverse effects and changes in HIV viral load.	also taking isoniazid for tuberculosis prophylaxis, dolutegravir exposure did not show a significant increase with the co-administration of dihydroartemisinin/piperaquine, unlike those not taking isoniazid.	exposure could have clinical implications over time. **Prior Use of Dihydroartemisinin/Piperaquine:** Participants had received dihydroartemisinin/piperaquine treatment six weeks before co-administration with dolutegravir, which might have introduced uncontrolled variability in the pharmacokinetics. However, the authors considered residual effects unlikely due to piperaquine's short half-life.
Impact of Dolutegravir-Based Antiretroviral Therapy on Piperaquine Exposure following Dihydroartemisinin-Piperaquine Intermittent Preventive Treatment of Malaria in Pregnant Women Living with HIV	The objective of the article is to investigate the impact of dolutegravir-based antiretroviral therapy on piperaquine exposure after intermittent preventive treatment with dihydroartemisinin-piperaquine for malaria in pregnant women living with HIV. The study aims to compare piperaquine plasma concentrations when coadministered with efavirenz-based therapy versus dolutegravir-based therapy, to evaluate whether the use of dolutegravir maintains the efficacy of antimalarial treatment in this group of women.	**Study Design:** An open-label, non-randomized, fixed-sequence pharmacokinetic study was conducted. The study compared piperaquine plasma concentrations in pregnant women living with HIV when co-administered with efavirenz-based antiretroviral therapy (ART) and then with dolutegravir-based ART. **Study Population:** The study included 13 pregnant women living with HIV in their second or third trimester of pregnancy. All participants were virologically suppressed and had been on efavirenz-based ART before switching to dolutegravir. **Treatment:** Participants received a three-day course of dihydroartemisinin-piperaquine for malaria prevention. During the	**Greater Piperaquine Exposure with Dolutegravir:** Compared to efavirenz-based therapy, coadministration of dihydroartemisinin-piperaquine with dolutegravir resulted in a 57% higher piperaquine exposure (AUC0–672h) with a geometric mean ratio (GMR) of 1.57 (90% confidence interval [CI]: 1.28 to 1.93). **Higher Plasma Concentrations:** Piperaquine concentrations on day 7 (Cday 7) were 63% higher when administered with dolutegravir compared to efavirenz (GMR, 1.63; 90% CI: 1.29 to 2.11), and day 28 concentrations (Cday 28) were nearly three times higher (GMR, 2.96; 90% CI: 2.25 to 4.07). **No Difference in Maximum Concentration (Cmax):** There were no significant differences in piperaquine's	**Small Sample Size:** The study included only 13 participants, which limits the generalizability of the results. Although the study achieved the necessary statistical power to detect changes in piperaquine exposure, a larger sample size would have provided greater robustness to the findings. **Fixed-Sequence Design:** By using a fixed-sequence design, where all participants switched from efavirenz to dolutegravir, the effect of time (e.g., pregnancy progression) on the results cannot be entirely ruled out. While regression analysis showed no significant effect of trimester on piperaquine exposure, this potential influence cannot be completely excluded with such a small sample size.

	first phase of the study, the treatment was co-administered with efavirenz. The women were then switched to dolutegravir and received the same antimalarial treatment again.<br>**Sampling and Analysis:** Intensive pharmacokinetic analyses were performed to measure piperaquine plasma concentrations at different time points: days 0, 7, 14, and 28. A non-compartmental analysis was used to calculate pharmacokinetic parameters such as area under the curve (AUC), maximum concentration (Cmax), and elimination half-life (t1/2).<br>**Comparison:** Data from the phase when participants were on efavirenz-based ART (sequence 1) were compared with data after switching to dolutegravir (sequence 3) to evaluate how piperaquine exposure varied between the two ART regimens.	maximum concentration (Cmax) between dolutegravir and efavirenz regimens (GMR, 1.09; 90% CI: 0.79 to 1.49).<br>**Safety and Tolerability:** The combination of dolutegravir and dihydroartemisinin-piperaquine was well tolerated, with no serious drug-related adverse events observed during the study.<br>**Clinical Implications:** The findings suggest that dolutegravir-based antiretroviral therapy would maintain the efficacy of dihydroartemisinin-piperaquine for malaria prevention in pregnant women living with HIV, without the need for dose adjustments.<br>**Impact of Isoniazid:** Post hoc analyses showed that piperaquine exposure was higher in participants not receiving isoniazid prophylaxis, a CYP3A4 inhibitor, although the difference was modest.	**Effect of ART Switch Mid-Study:** Due to changes in national health policy, the women were switched from efavirenz to dolutegravir before completing the efavirenz treatment phase. This may have underestimated the full impact of efavirenz on piperaquine exposure, particularly in calculating the AUC up to day 28.<br>**Inconclusive Impact of Isoniazid:** Only 4 participants were receiving isoniazid prophylaxis, which limited the study's ability to accurately assess the impact of this medication on piperaquine exposure. Further studies are needed to investigate this effect.<br>**Clinically Relevant Findings:** Despite the small sample size, the findings suggest that coadministration of dolutegravir does not reduce the efficacy of dihydroartemisinin-piperaquine, which is a clinically relevant outcome for malaria prevention in pregnant women living with HIV. However, the low percentage of women achieving target piperaquine concentrations on day 28 (30% with dolutegravir and 0% with efavirenz) may indicate that adjustments to the dosing regimen are needed to ensure adequate protection during pregnancy.

Safety and pharmacokinetics of dolutegravir in pregnant mothers with HIV infection and their neonates: A randomised trial (DolPHIN-1 study)	To investigate the safety and pharmacokinetics of dolutegravir (DTG) in pregnant women with HIV, specifically during the third trimester of pregnancy, as well as in their newborns. Additionally, the impact of DTG on viral suppression was compared to standard treatment with efavirenz (EFV). The study also examined the transfer of the drug across the placenta and into breast milk, aiming to determine whether the use of DTG in women initiating treatment during the third trimester could reduce the risk of vertical transmission of HIV.	The study was an open-label, randomized clinical trial comparing dolutegravir (DTG)-based treatment with standard efavirenz (EFV)-based treatment in HIV-positive pregnant women who initiated treatment in the third trimester of pregnancy.<br>Sixty previously untreated HIV-positive pregnant women between 28 and 36 weeks' gestation were included. Participants were recruited from Uganda and South Africa.<br>Women were randomized in a 1:1 ratio to receive DTG (50 mg once daily) or EFV as part of their antiretroviral therapy (ART). All treatments included tenofovir disoproxil fumarate and lamivudine or emtricitabine.<br>Intensive sampling was conducted to assess DTG levels in maternal plasma during the third trimester and after delivery, as well as in umbilical cord blood, breast milk and infant plasma. The concentration ratio between maternal blood and cord blood, breast milk and infant plasma was measured.<br>Viral load levels were monitored at different time points (day 14, after two weeks postpartum and through six months postpartum) to assess the efficacy of	**Viral suppression:** The time to reach viral suppression (viral load <50 copies/mL) was significantly faster in the dolutegravir-treated group (DTG) compared with the efavirenz-treated group (EFV). The median time to reach undetectable viral load was 32 days in the DTG group compared to 72 days in the EFV group.<br>At 2 weeks postpartum, 69% of women in the DTG group had a viral load <50 copies/mL, compared with only 39% in the EFV group.<br>**Pharmacokinetics:**Although some women in the DTG group had lower than expected dolutegravir concentrations during the third trimester, exposure to the drug was sufficient to achieve viral suppression in most cases.<br>Significant transfer of dolutegravir across the placenta (121% of maternal plasma concentrations) and moderate passage through breast milk (3% of maternal plasma concentrations) were observed. DTG levels in infants remained elevated during the first two weeks of life due to slow elimination of the drug.<br>**Safety:** Both treatments (DTG and EFV) were well tolerated in mothers, with a similar frequency of adverse events. No significant differences were	Due to national health policies in Uganda and South Africa, pregnant women with HIV were started on an efavirenz (EFV)-based regimen immediately after HIV diagnosis, before being randomized to continue efavirenz or switch to dolutegravir (DTG). This means that all participants had initial exposure to EFV, which may have attenuated the differences observed between treatments early in the trial.<br>The study included only 60 women, which limits the generalizability of the findings. In addition, the analysis of pharmacokinetics in neonates was based on a small number of cases, which could have influenced the accuracy of the results on neonatal exposure to dolutegravir.<br>DTG treatment was discontinued two weeks after delivery. This meant that the long-term safety of prolonged DTG exposure in neonates and possible effects on infant development could not be fully assessed, as follow-up was limited in time.

		treatment in reducing HIV viral load. Maternal and neonatal safety assessments were performed, including follow-up of adverse events, obstetric outcomes, congenital anomalies and neonatal outcomes.	observed in terms of obstetric or neonatal complications between the groups. No congenital malformations were recorded in the newborns of the DTG group, whereas two congenital malformations were observed in the EFV group, although neither was considered treatment-related. **Neonatal outcomes:** Neonates born to DTG-treated mothers had no significant drug-related problems. Considerable exposure to DTG was observed in neonates due to placental transfer and lactation, but no serious adverse effects associated with this exposure were identified.	
Dolutegravir twice-daily dosing in children with HIV-associated tuberculosis: a pharmacokinetic and safety study within the open-label, multicentre, randomised, non-inferiority ODYSSEY trial	To evaluate the safety and pharmacokinetics of dolutegravir dosed twice daily in children with HIV-associated tuberculosis who are receiving rifampicin, a drug that can reduce plasma concentrations of several antiretrovirals.	The study was conducted as part of the ODYSSEY trial, an open-label, multicenter, randomized, controlled study with a pharmacokinetic substudy. Children who received dolutegravir twice daily along with rifampicin were monitored to compare the pharmacokinetic parameters of dolutegravir with and without rifampicin. A 12-hour pharmacokinetic profile was conducted with dolutegravir twice daily and rifampicin, and a 24-hour profile was conducted with dolutegravir once daily.	Twice-daily dosing of dolutegravir in children with HIV-associated tuberculosis was safe and effective in overcoming the inducing effect of rifampicin. No serious adverse events attributable to dolutegravir were identified, suggesting it is a viable option for this population. Plasma concentrations of dolutegravir in all children treated with rifampicin and dolutegravir twice daily exceeded the effective threshold of 90% (0.32 mg/L), ensuring that concentrations were sufficient to suppress HIV. During follow-up, 15 serious adverse events	A limitation was the low number of younger children in the study. No serious adverse events related to dolutegravir were identified, although some children experienced events such as immune reconstitution inflammatory syndrome (TB-IRIS). It was noted that rifampicin levels were lower than optimal in several children, which could suggest the need to adjust rifampicin doses in treatment.

			occurred in 11 children (30%), including two deaths, although none of the serious adverse events were considered related to dolutegravir treatment. The majority of children achieved virological suppression.	
Raltegravir pharmacokinetics during pregnancy	The main objective of the study was to evaluate the pharmacokinetics (PK) of raltegravir in HIV-infected women during pregnancy and the postpartum period. The study aimed to determine whether drug exposure levels during pregnancy were comparable to those observed in non-pregnant women, in order to guide dosage recommendations for this special population.	The study is part of the IMPAACT 1026s protocol, a multicenter, prospective trial that evaluated the pharmacokinetics of antiretrovirals in pregnant women with HIV.<br>A total of 42 pregnant women receiving raltegravir (400 mg twice daily) as part of their routine HIV treatment were included.<br>Intensive 12-hour pharmacokinetic profiles were conducted to assess drug concentration during the second and third trimesters of pregnancy, and again between 6 and 12 weeks postpartum.<br>Data were collected on pre-dose plasma concentrations (C12h), maximum concentration (Cmax), and area under the curve (AUC) to measure overall drug exposure.<br>Maternal and umbilical cord plasma concentrations were also measured at the time of delivery to evaluate the placental transfer of the drug.	A significant reduction in raltegravir exposure was observed during pregnancy. The AUC was approximately 50% lower during pregnancy compared to the postpartum period, with medians of 6.6 μghr/mL (second trimester), 5.4 μghr/mL (third trimester), and 11.6 μg*hr/mL (postpartum).<br>Raltegravir concentrations showed high variability, but the minimum concentrations (C12h) exceeded the target of 0.035 μg/mL in 69%, 80%, and 79% of women in the second trimester, third trimester, and postpartum, respectively.<br>High placental transfer was observed, with a cord-to-maternal concentration ratio of 1.5, suggesting that the drug crosses the placenta effectively.<br>92% of the women achieved viral suppression, with HIV RNA levels below 400 copies/mL at delivery, and no infants born to these women were infected with HIV.	Methodological strengths: The study was multicenter, allowing for the inclusion of a diverse population of women, and employed a robust pharmacokinetic approach with intensive sampling.<br>Serious methodological limitations: A major limitation was the high variability in raltegravir levels among women, which could complicate the extrapolation of results to the general population of pregnant women with HIV. Additionally, while the sample size was adequate, it did not allow for the detection of rare adverse events related to raltegravir use during pregnancy.<br>Irrelevant findings: The relationship between raltegravir levels and virological response was inconclusive, consistent with findings in non-pregnant adults, suggesting that plasma levels of raltegravir do not clearly predict virological response.
Raltegravir Pharmacokinetics in Neonates Following Maternal Dosing	The objective of the article is to describe the pharmacokinetics of the "washout" phase	**Study Design:**<br>The IMPAACT P1097 study was a multicenter trial designed to	**Placental Transfer of Raltegravir:**<br>• Raltegravir crossed the placenta	**Limitations in Pharmacokinetic Sampling Times:**<br>The study did not

	and the safety of prenatal and perinatal exposure to raltegravir in neonates born to mothers living with HIV who received raltegravir-based antiretroviral therapy during pregnancy. The study aims to understand how raltegravir, acquired through placental transfer, is eliminated in newborns and the potential associated risks, such as bilirubin toxicity and the development of viral resistance.	describe the elimination pharmacokinetics of raltegravir and the safety of prenatal/intrapartum exposure in neonates born to pregnant women living with HIV who received raltegravir-based antiretroviral therapy.<br>**Participants:**<br>● The study included 22 mother-infant pairs, with pharmacokinetic data available for 19 pairs.<br>● Mothers were required to have received raltegravir (400 mg twice daily) for at least two weeks prior to delivery.<br>**Samples and Data Collection:**<br>● Maternal plasma and umbilical cord blood samples were collected at delivery to measure raltegravir concentrations.<br>● Neonatal plasma samples were taken at specific intervals (1–5, 8–14, 18–24, and 30–36 hours after birth).<br>● Physical examinations of the neonates were conducted shortly after birth.<br>**Genotyping:**<br>● UGT1A1 genotyping was performed on 17 of the 22 neonates to investigate the relationship between polymorphisms in this enzyme and raltegravir elimination.<br>**Data Analysis:**<br>● Descriptive statistics were used to analyze raltegravir concentrations in maternal	efficiently, with a median concentration in umbilical cord blood of 957 ng/mL, higher than the maternal plasma concentration (median 540 ng/mL). The mean cord-to-maternal concentration ratio was 1.48.<br>**Prolonged Elimination in Neonates:**<br>● Raltegravir elimination in neonates was highly variable and, in some cases, prolonged. The median elimination half-life (t½) was 26.6 hours, ranging from 9.3 to 184 hours.<br>● Nearly half of the neonates experienced an increase in raltegravir concentration during the first 12–24 hours after birth, despite not receiving additional doses postnatally. This is likely attributed to enterohepatic recirculation.<br>**Sustained Concentrations:**<br>● In most neonates, raltegravir concentrations remained above the inhibitory concentration 95 (IC95) for wild-type HIV (14 ng/mL) up to 30–36 hours after birth.<br>**Toxicity and Safety:**<br>● Five neonates (22.7%) experienced grade 3 or 4 laboratory events, including elevated bilirubin, high creatinine, and decreased hemoglobin, among others. However, none of these events were related to	anticipate the prolonged half-life of raltegravir in some neonates. As a result, the sampling period was limited, as samples were collected over a short timeframe before the neonates were discharged from the hospital. This restricted a comprehensive evaluation of the drug's elimination in certain cases.<br>**Absence of Direct Raltegravir Administration to Neonates:**<br>The study focused solely on transplacental exposure to raltegravir and did not assess the drug's absorption and elimination when administered directly to neonates. Consequently, there is no data on direct administration in newborns, limiting the applicability of the findings to clinical scenarios involving direct treatment.<br>**Small Sample Size:**<br>Although 22 mother-infant pairs were included in the study, only 19 pairs had complete pharmacokinetic data. The small sample size may limit the generalizability of the findings to a broader neonatal population.<br>**Lack of Long-Term Follow-Up:**<br>While the study included a follow-up period of 20 weeks, it does not provide information on the potential long-term effects of raltegravir exposure in

		plasma, umbilical cord blood, and neonatal plasma.<br>● Regression analysis was employed to estimate the elimination half-life of raltegravir in neonates.<br>**Follow-Up:**<br>● Neonates were monitored for up to 20 weeks after birth to detect any signs of raltegravir-related toxicity.	maternal raltegravir use.<br>● Only one neonate required phototherapy for hyperbilirubinemia, and there were no reports of deaths or stillbirths.<br>**UGT1A1 Genotypes:**<br>● No significant differences in raltegravir elimination or plasma concentrations were observed between neonates with the UGT1A1 (TA)6/(TA)6 genotype (wild-type allele) and those with other genotype variants.	neonates. Long-term data would be crucial to assess potential late-onset effects of the drug.<br>**Variability in Raltegravir Elimination:** There was significant variability in raltegravir elimination among neonates, suggesting that additional factors, such as hepatic development and UGT1A1 enzyme function, may play a significant role. However, the study did not fully explore the causes of this variability, leaving open questions about other contributing factors.
A study of the pharmacokinetics, safety, and efficacy of bictegravir/emtricitabine/tenofovir alafenamide in virologically suppressed pregnant women with HIV	The main objective of the study was to evaluate the pharmacokinetics, safety, and efficacy of the once-daily combination of bictegravir/emtricitabine/tenofovir alafenamide (B/F/TAF) in pregnant women with HIV-1 who were virologically suppressed. The study aimed to confirm whether the standard dose of B/F/TAF was adequate during pregnancy, despite the physiological changes that could affect pharmacokinetics.	The study was a phase 1b, open-label, multicenter, single-arm trial. A total of 33 virologically suppressed pregnant women (with HIV-1 viral load <50 copies/mL) participated, receiving the treatment from the second or third trimester until approximately 16 weeks postpartum. Maternal plasma samples were collected during the second and third trimesters, as well as at 6 and 12 weeks postpartum, to evaluate the concentrations of BIC, FTC, and TAF. Neonates were also assessed with sporadic pharmacokinetic testing and HIV transmission monitoring up to 4–8 weeks after birth.	Exposure to BIC, FTC, and TAF was significantly lower during pregnancy compared to the postpartum period. Nevertheless, BIC concentrations remained above the level required to maintain virological suppression ($paEC_{95}$).<br>All participants maintained virological suppression throughout pregnancy and up to the end of the follow-up, with no instances of virological failure or the emergence of treatment resistance.<br>No cases of perinatal HIV transmission were reported among the 29 women who completed the study.<br>B/F/TAF was well tolerated, with most adverse events classified as mild or moderate. There were no	Methodological shortcomings: The study was open-label and lacked a control group, limiting the ability to make definitive comparisons regarding efficacy and safety against other therapeutic alternatives. Additionally, the sample size was relatively small, which could limit the generalizability of the results.<br>Irrelevant findings: The study did not find significant differences in safety or efficacy compared to previous studies, which was expected, but it reinforces the lack of need to adjust the dose of B/F/TAF in pregnant women.

			treatment interruptions due to severe drug-related adverse events.	

Entry and Fusion Inhibitors

Pharmacokinetics and their rare but crucial applications during pregnancy

1.Article	2. Objective	3. Methodological aspects	4. Results	5. Additional observations
Elvitegravir/cobicistat pharmacokinetics in pregnant and postpartum women with HIV	The main objective of the study was to evaluate the pharmacokinetics of darunavir and cobicistat during pregnancy compared to the postpartum period, as well as to analyze the exposure of newborns to these drugs through washout samples collected after birth. The aim was to determine whether standard doses of these medications provided adequate exposure to prevent virologic failure and perinatal HIV transmission.	The study was an open-label, non-randomized, multicenter phase IV clinical trial conducted at various institutions in the United States.<br>A total of 29 pregnant women with HIV participated, receiving a fixed-dose combination of 800 mg of darunavir and 150 mg of cobicistat once daily.<br>Intensive 24-hour pharmacokinetic profiles were collected during the second and third trimesters of pregnancy and the postpartum period.<br>Liquid chromatography-tandem mass spectrometry (LC-MS/MS) assays were used to measure darunavir and cobicistat concentrations in plasma.	The exposure levels of darunavir and cobicistat were significantly lower during pregnancy compared to the postpartum period.<br>**Darunavir:** The AUC was 53% lower in the second trimester and 56% lower in the third trimester.<br>**Cobicistat:** The AUC was 50% lower in the second trimester and 56% lower in the third trimester.<br>The placental transfer of both drugs was minimal. Most newborns did not have measurable concentrations of the drugs in their plasma, indicating low fetal exposure.<br>It was observed that while most women maintained viral suppression, some experienced adverse events such as preterm labor, potentially related to the treatment.	The lack of a control group or randomization limits the ability to make more rigorous comparisons.<br>The selection of women who were already receiving the treatment may introduce bias, as only those responding favorably to the treatment were included. This could have overestimated positive outcomes and underestimated adverse events.<br>Washout samples from newborns were limited, and the sampling time points were broad, which may have impacted the precise assessment of drug elimination in neonates.<br>The results may be difficult to generalize due to these methodological limitations.
